# Efficacy of Pharmacist Based Diabetes Educational Interventions on Clinical Outcomes of Adults With Type 2 Diabetes Mellitus: A Network Meta-Analysis

**DOI:** 10.3389/fphar.2018.00339

**Published:** 2018-04-10

**Authors:** Allah Bukhsh, Tahir M. Khan, Shaun W. H. Lee, Learn-Han Lee, Kok-Gan Chan, Bey-Hing Goh

**Affiliations:** ^1^School of Pharmacy, Monash University, Jalan Lagoon Selatan, Bandar Sunway, Malaysia; ^2^Institute of Pharmaceutical Sciences, University of Veterinary and Animal Sciences, Lahore, Pakistan; ^3^Asian Centre for Evidence Synthesis in Population, Implementation and Clinical Outcomes, Health and Well-being Cluster, Global Asia in the 21st Century Platform, Monash University Malaysia, Bandar Sunway, Malaysia; ^4^Biofunctional Molecule Exploratory Research Group, School of Pharmacy, Monash University Malaysia, Bandar Sunway, Malaysia; ^5^Novel Bacteria and Drug Discovery Research Group, School of Pharmacy, Monash University Malaysia, Bandar Sunway, Malaysia; ^6^Center of Health Outcomes Research and Therapeutic Safety, School of Pharmaceutical Sciences, University of Phayao, Phayao, Thailand; ^7^International Genome Centre, Jiangsu University, Zhenjiang, China; ^8^Division of Genetics and Molecular Biology, Institute of Biological Sciences, Faculty of Science, University of Malaya, Kuala Lumpur, Malaysia

**Keywords:** diabetes education, glycosylated hemoglobin, type 2 diabetes mellitus, pharmaceutical care, meta-analysis

## Abstract

**Background:** Comparative efficacy of different pharmacist based interventions on glycemic control of type 2 diabetes patients is unclear. This review aimed to evaluate and compare the efficacy of different pharmacist based interventions on clinical outcomes of type 2 diabetes patients.

**Methods:** A systematic search was conducted across five databases from date of database inception to September 2017. All randomized clinical trials evaluating the efficacy of pharmacist based interventions on type 2 diabetes patients were included for network meta-analysis (NMA). The protocol is available with PROSPERO (CRD42017078854).

**Results:** A total of 43 studies, involving 6259 type 2 diabetes patients, were included. NMA demonstrated that all interventions significantly lowered glycosylated hemoglobin (HbA1c) levels compared to usual care, but there was no statistical evidence from this study that one intervention was significantly better than the other for reducing HbA1c levels. Pharmacist based diabetes education plus pharmaceutical care showed maximum efficacy for reducing HbA1c levels [−0.86, 95% CI −0.983, −0.727; *p* < 0.001]. Pharmacist based diabetes education plus pharmaceutical care was observed to be statistically significant in lowering levels of systolic blood pressure [−4.94; 95%CI −8.65, −1.23] and triglycerides levels [−0.26, 95%CI −0.51, −0.01], as compared to the interventions which involved diabetes education by pharmacist, and for body mass index (BMI) [−0.57; 95%CI −1.25, −0.12] in comparison to diabetes education by health care team involving pharmacist as member.

**Conclusion:** The findings of this review demonstrate that all interventions had a significantly positive effect on HbA1c, but there was no statistical evidence from this study that one intervention was significantly better than the other for achieving glycemic control.Pharmacist based diabetes education plus pharmaceutical care showed maximum efficacy on HbA1c and rest of the clinical outcomes.

## Introduction

Type 2 diabetes mellitus, a chronic metabolic disorder, if poorly controlled, results in microvascular and macrovascular complications (DeCoster, 2001). Globally 415 million people have been diagnosed with diabetes and this number is projected to rise to 642 million by 2040 (Atlas IDFI, 2016). Despite the benefits of anti-hyperglycemic drugs, literature indicates poor achievement of desired therapeutic outcomes in patients with type 2 diabetes (Collins et al., 2003; García-Pérez et al., 2013). Non-adherence to medication and recommended life style are major barriers to ideal glycemic control (HbA1c < 7%) in chronic type 2 diabetes patients (Ali et al., 2013; García-Pérez et al., 2013; Lee et al., 2016).

Adherence to self-management practices (healthy diet, regular exercise, self-monitoring of blood glucose, and proper use of medication) is considered to play pivotal role in achieving euglycaemia in chronic type 2 diabetes patients (Compeán Ortiz et al., 2010; Inzucchi et al., 2012; Ahola and Groop, 2013; Lee et al., 2016). Pharmacists are playing a key role in providing self-management education to diabetes patients. Literature indicates a number of interventional studies involving pharmacist based interventions, showing clinically significant improvements in the clinical outcomes of the diabetes patients (Machado et al., 2007; Wubben and Vivian, 2008; Pousinho et al., 2016; Van Eikenhorst et al., 2017; Yaghoubi et al., 2017).

To date several systematic reviews and meta-analysis have been published evaluating the impact of pharmacist based interventions in diabetes patients with respect to usual care (Machado et al., 2007; Wubben and Vivian, 2008; Pousinho et al., 2016; Van Eikenhorst et al., 2017; Yaghoubi et al., 2017). However, there is no study which had compared and presented that which pharmacist based intervention is better than the other, with statistical evidence. Although systematic reviews and pairwise meta-analysis are important tools of policy makers for devising guidelines and clinical protocols, but they produce partial information, because among many of the available interventions only few are examined in head-to-head comparisons (Greco et al., 2015; Tonin et al., 2017). Network meta-analysis (NMA) has an advantage to make quantitative comparison of the interventions that have not been compared directly in the studies (Greco et al., 2015). Our NMA will facilitate policy makers to tailor their choice of intervention for achieving desired clinical outcomes in type 2 diabetes patients, keeping in view the maximum utilization of the available resources in a local healthcare context.

In this study, a NMA was performed to determine the relative efficacy of various pharmacist based interventions involving diabetes education alone and in combination with pharmaceutical care, and those interventions in which diabetes education was provided by health care team including pharmacist as team member, on clinical outcomes of the type 2 diabetes patients. We choose to use glycosylated hemoglobin (HbA1c) as primary outcome, as it has been shown to be a good surrogate marker for diabetes related complications (DeCoster, 2001). Other secondary outcomes include fasting blood sugar (FBS), body mass index (BMI), blood pressure control and lipid profile.

## Methodology

### Data sources and search strategy

Five electronic databases (PubMed, ProQuest, Scopus, EBSCOhost, and Ovid) were searched from date of database inception to September 2017. The PubMed search strategy served as a reference for the development of search strategies for the remaining databases. The search terms used in this review, included medical subject headings [MeSH] and text terms combined with Boolean operators. The strategic search terms were; “Diabetes Mellitus, Type 2” OR T2DM OR “Non-insulin dependent diabetes mellitus” OR “NIDDM” OR “Type 2 diabetes” AND “Pharmaceutical care” OR “Clinical pharmacy” OR “Community pharmacy” OR Pharmacist^*^ OR “Pharmaceutical services” OR Education OR Intervention^*^ OR “Self care” OR “Self-management” OR “Medication Management” AND “Knowledge” OR “Hemoglobin A, Glycosylated” OR “HbA1c” OR “glycemic control” OR “Behavior change.” The detailed search strategy used for each database is provided in the Appendix I of the Supplementary file.

### Inclusion criteria

Studies were included in this review if they were: (1) randomized controlled trials or cluster-randomized controlled trials; (2) evaluating the efficacy of educational interventions (with or without pharmaceutical care planning) delivered by pharmacists alone or in collaboration with other health care professionals; (3) directed at patients with type 2 diabetes only; (4) reporting glycosylated hemoglobin as primary clinical outcome (alone or in combination with any of the other clinical outcomes, such as FBS, BMI, lipid profile, and blood pressure); (5) conducted in community pharmacy, outpatient primary care or hospital settings; (6) published as an original study in a peer-reviewed journal; and (7) available as full text in English language. Conference abstracts, review articles and non-RCT studies were excluded.

### Study selection

Two reviewers independently screened all titles and abstracts, retrieved from the electronic databases using the defined selection criteria. Then, the full text of each potentially eligible article was obtained and screened independently by two reviewers to further assess its suitability for inclusion in this review. Any disagreement was resolved through discussion.

### Data extraction and synthesis

A single reviewer extracted data from included studies using a standardized form. Subsequently, another reviewer independently checked the extracted data. The data extracted from each study included authors, title, publication year, study design, setting and country where the study took place, sample size, patient age and gender, follow-up duration, details of pharmacist interventions and usual care, inclusion and exclusion criteria, and study outcomes. The results for the outcome measures included in this review were summarized as change from baseline to final follow-up in intervention and control groups.

### Risk of bias assessment

Two reviewers independently assessed the risk of bias of the included studies using the Cochrane risk of bias tool. For RCTs, each risk of bias item was rated as “low risk” if it was unlikely that a bias would seriously alter the results; “unclear” if it was likely that a bias would raise some doubt about the results; or “high risk” if it was likely that a bias would seriously alter the results. Any disagreement was resolved through discussion.

### Data analysis

Meta-analysis and NMA were performed by using STATA version 14. Mean difference was estimated to calculate the overall comparative efficacy of all interventions using random effect model. All the *p*-values were set to be < 0.05 with 95% confidence intervals (according to whether the confidence interval included the null value) to assess significance.

Sub-group analysis were performed for primary and secondary clinical outcomes, for different interventions, so as to explain the heterogeneity among the studies. Sensitivity analysis were done to check the robustness of the results by performing sub-group analysis regarding baseline HbA1c levels (< 8% and more or equal to 8%), duration of interventions, geographical areas where the studies were performed and their influence on primary clinical outcome (HbA1c). In addition, pairwise comparison for the treatment effect was carried out to generate the forest plot for the NMA. League tables were generated using treatment effect, mean difference (MD; 95% confidence interval) for all direct and indirect effects of the various interventions. Relative efficacy of different interventions for primary outcome (HbA1c) was evaluated by using surface under the cumulative ranking curve (SUCRA) and mean ranks.

### Study protocol registration

The study protocol is registered with PROSPERO (Registration No. CRD42017078854).

## Results

**Figure 1:**
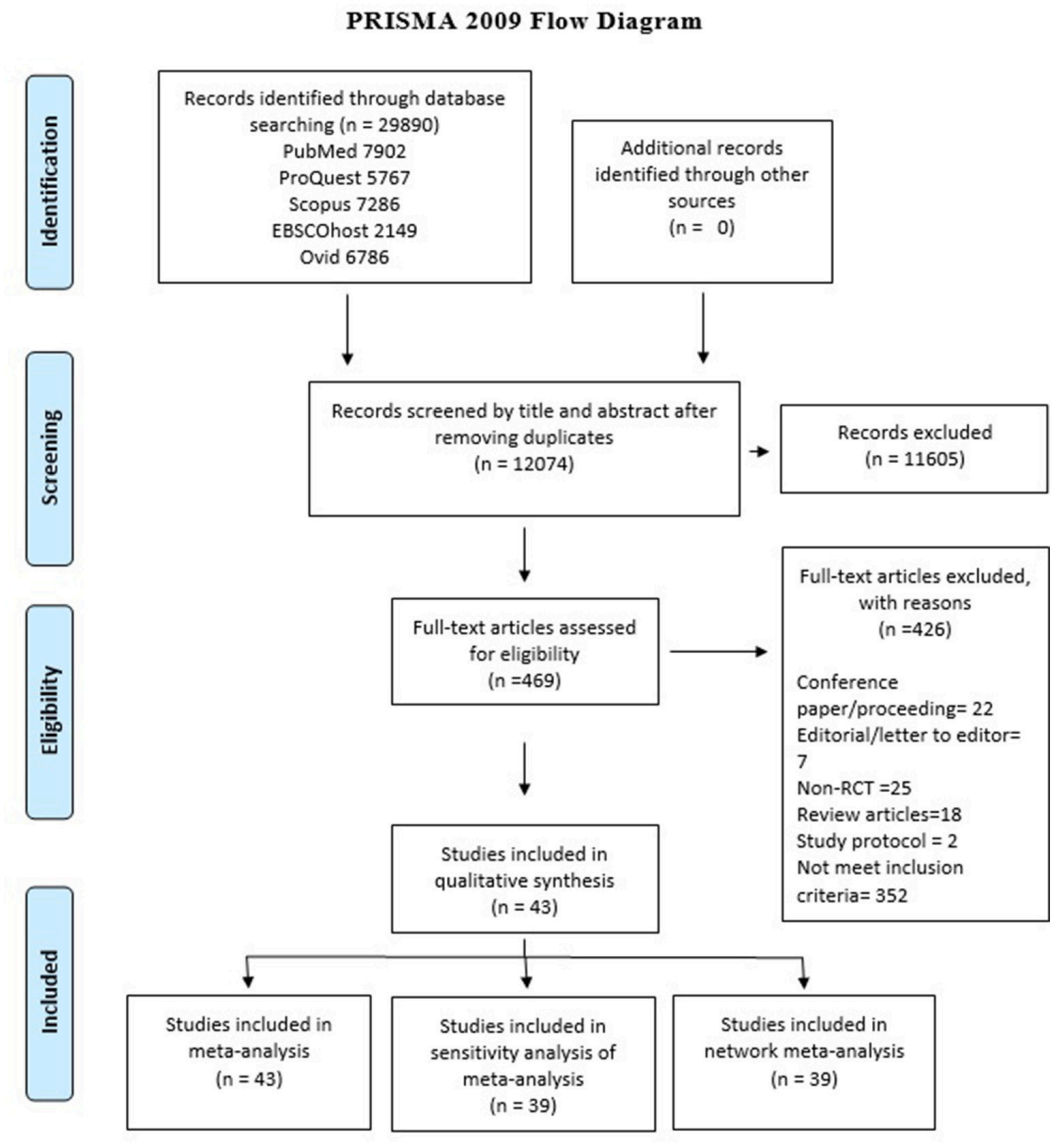
PRISMA flow diagram of study selection.

In total 29,890 articles were identified from the electronic database searches, after removing duplication (*n* = 17,816), the final count reduced to 12,074. On the basis of title and abstract evaluation 11,605 studies were excluded as they did not meet the inclusion criteria of the study. Full text assessment was carried out for 469 studies by two reviewers and 43 studies were finally included for qualitative and quantitative review, the details are presented in PRISMA flow diagram of Figure 1. Reasons for exclusion after full-text assessment are presented in Supplementary Table 1.

### Description of included studies

Among the studies included in this review, *n* = 3 were cluster-randomized controlled trials, (Armour et al., 2004; Krass et al., 2007; Mehuys et al., 2011) whereas rest of the studies, *n* = 40 were parallel randomized controlled trials (Sarkadi and Rosenqvist, 2004; Clifford et al., 2005; Hayward et al., 2005; Odegard et al., 2005; Rothman et al., 2005; Suppapitiporn et al., 2005; Taylor et al., 2005; Fornos et al., 2006; Scott et al., 2006; Ko et al., 2007; Al Mazroui et al., 2009; Doucette et al., 2009; Jameson and Baty, 2010; Kang et al., 2010; Taveira et al., 2010; Cohen et al., 2011; Farsaei et al., 2011; Sriram et al., 2011; Ali et al., 2012; Chan et al., 2012; Jacobs et al., 2012; Jarab et al., 2012; Mahwi and Obied, 2013; Mourão et al., 2013; Samtia et al., 2013; Castejón et al., 2014; Chung et al., 2014; Ahmad et al., 2015; Cani et al., 2015; Chow et al., 2015; Jahangard-Rafsanjani et al., 2015; Wishah et al., 2015; Butt et al., 2016; Chen et al., 2016; Lim et al., 2016; Nascimentoa et al., 2016; Tourkmani et al., 2016; Korcegez et al., 2017; Shao et al., 2017; Siaw et al., 2017). Most of the included studies (*n* = 20) were conducted in Asia (Suppapitiporn et al., 2005; Ko et al., 2007; Kang et al., 2010; Farsaei et al., 2011; Sriram et al., 2011; Chan et al., 2012; Jarab et al., 2012; Mahwi and Obied, 2013; Samtia et al., 2013; Chung et al., 2014; Chow et al., 2015; Jahangard-Rafsanjani et al., 2015; Wishah et al., 2015; Butt et al., 2016; Chen et al., 2016; Lim et al., 2016; Tourkmani et al., 2016; Shao et al., 2017; Siaw et al., 2017), followed by North America (*n* = 11) (Hayward et al., 2005; Odegard et al., 2005; Rothman et al., 2005; Scott et al., 2006; Doucette et al., 2009; Jameson and Baty, 2010; Taveira et al., 2010; Cohen et al., 2011; Jacobs et al., 2012; Castejón et al., 2014; Korcegez et al., 2017), Europe (*n* = 5) (Sarkadi and Rosenqvist, 2004; Fornos et al., 2006; Mehuys et al., 2011; Ali et al., 2012; Nascimentoa et al., 2016), Australia (*n* = 4) (Armour et al., 2004; Clifford et al., 2005; Taylor et al., 2005; Krass et al., 2007), South America (*n* = 2) (Mourão et al., 2013; Cani et al., 2015), and Africa (*n* = 1) (Ahmad et al., 2015).

Seven studies has duration of intervention was < 6 months (Taveira et al., 2010; Farsaei et al., 2011; Mahwi and Obied, 2013; Samtia et al., 2013; Castejón et al., 2014; Jahangard-Rafsanjani et al., 2015; Lim et al., 2016) 14 studies had 6 months (Hayward et al., 2005; Krass et al., 2007; Cohen et al., 2011; Mehuys et al., 2011; Jarab et al., 2012; Mourão et al., 2013; Ahmad et al., 2015; Cani et al., 2015; Chow et al., 2015; Wishah et al., 2015; Chen et al., 2016; Nascimentoa et al., 2016; Shao et al., 2017; Siaw et al., 2017) and 22 studies' duration of intervention was more than 6 months (Armour et al., 2004; Sarkadi and Rosenqvist, 2004; Clifford et al., 2005; Odegard et al., 2005; Rothman et al., 2005; Taylor et al., 2005; Fornos et al., 2006; Scott et al., 2006; Ko et al., 2007; Johnson et al., 2008; Al Mazroui et al., 2009; Doucette et al., 2009; Jameson and Baty, 2010; Kang et al., 2010; Cohen et al., 2011; Sriram et al., 2011; Ali et al., 2012; Chan et al., 2012; Jacobs et al., 2012; Chung et al., 2014; Butt et al., 2016; Chen et al., 2016; Tourkmani et al., 2016; Korcegez et al., 2017). Pharmacist provided only educational interventions without pharmaceutical care in, *n* = 17 studies (Sarkadi and Rosenqvist, 2004; Hayward et al., 2005; Suppapitiporn et al., 2005; Krass et al., 2007; Al Mazroui et al., 2009; Farsaei et al., 2011; Mehuys et al., 2011; Sriram et al., 2011; Chan et al., 2012; Samtia et al., 2013; Castejón et al., 2014; Chow et al., 2015; Jahangard-Rafsanjani et al., 2015; Butt et al., 2016; Lim et al., 2016; Tourkmani et al., 2016; Shao et al., 2017) or as a member of health care team in, *n* = 4 studies (Ko et al., 2007; Kang et al., 2010; Taveira et al., 2010; Siaw et al., 2017). In rest of the included studies (*n* = 22) pharmacist provided diabetes education in combination with pharmaceutical care (Armour et al., 2004; Clifford et al., 2005; Odegard et al., 2005; Rothman et al., 2005; Taylor et al., 2005; Fornos et al., 2006; Scott et al., 2006; Doucette et al., 2009; Jameson and Baty, 2010; Cohen et al., 2011; Ali et al., 2012; Jacobs et al., 2012; Jarab et al., 2012; Mahwi and Obied, 2013; Mourão et al., 2013; Chung et al., 2014; Ahmad et al., 2015; Cani et al., 2015; Wishah et al., 2015; Chen et al., 2016; Nascimentoa et al., 2016; Korcegez et al., 2017) (detail for contents of pharmaceutical care intervention for individual study, is presented in Supplementary Table 2), in a varied healthcare settings, such as primacy care clinics, community pharmacies and hospital settings. The control group in the included studies received usual care.

The nature of intervention provided by pharmacist varied among the included studies and covered one or more of the following topics: education about diabetes and its complications, self-management education, education on medication use, medication adherence counseling, pharmaceutical care planning, provision of free glucometer and pill counter, education on life style modification, education on self-monitoring of blood glucose (SMBG), and, provision of written educational material to type 2 diabetes patients. Overall, *n* = 6259 participants with type 2 diabetes, were involved in the included studies (*n* = 43). The follow-up duration of the included studies varied from 3 to 48 months. The detailed characteristics of the included studies are shown in Table 1.

**Table 1:** Characteristics of the included studies.

**Author, Year, Country**	**Study design, duration of study (months)**	**No. of type 2 diabetes patients (*n*), Lost to follow-up (*n*), mean age in years (*SD*), Gender (%) Female, Duration of type 2 diabetes in years (*SD*)**	**Pharmacist intervention**	**Control**	**Characteristics of pharmacist intervention**									
					**Education about diabetes & its complications**	**Self- management education**	**Counseling for medication use**	**Medication adherence counseling**	**Pharmaceutical Care**	**Provision of written educational material**	**Provision of free glucometer &/pill counter**	**Education on life style modification**	**Provision of SMBG data entry log book**	**SMBG education**
(Ahmad et al., 2015), Sudan	Parallel RCT, 6	No. of Patients (IG/CG): 200/100 Loss to follow-up (IG/CG): 3/3 Age (IG/CG): not mentioned Gender (IG/CG) female (%): 39.5/41 Duration of T2DM (IG/CG):	Pharmacist-led pharmaceutical care	Usual care	x		x		x			x		x
(Al Mazroui et al., 2009), United Arab Emirates	Parallel RCT, 12	No. of Patients (IG/CG): 120/120 Loss to follow-up (IG/CG): 3/3 Age (IG/CG): 48.7 (8.2) /49.9 (8.3) Gender (IG/CG) female (%): 30/31.7 Duration of T2DM (IG/CG):6.1 (2.9)/6.2 (2.7)	Pharmaceutical care programme	Usual care from medical and nursing staff	x		x	x		x		x		x
(Ali et al., 2012), United Kingdom	Parallel RCT, 12	No. of Patients (IG/CG): 25/23 Loss to follow-up (IG/CG): 2/0 Age (IG/CG): 66.4 (12.7)/66.8 (10.2) Gender (IG/CG) female (%): 56.5/43.5 Duration of T2DM (IG/CG):7.5 (4.8)/6.8 (3.5)	Pharmaceutical care	Usual care					x			x		
(Armour et al., 2004), Australia	Cluster RCT, 9	No. of Patients (IG/CG): 106/82 Loss to follow-up (IG/CG): not mentioned Age (IG/CG): 64 (9) / 65 (10) Gender (IG/CG) female (%): 65/49 Duration of T2DM (IG/CG): Not mentioned	Community Pharmacist delivered interventions		x	x	x	x		x				
(Butt et al., 2016), Malaysia	Parallel RCT, 6	No. of Patients (IG/CG): 33/33 Loss to follow-up (IG/CG): 4/3 Age (IG/CG): 57.42 ± 7.17/57.12 ± 10.78 Gender (IG/CG): 60.6/57.6 Duration of T2DM (IG/CG): not mentioned	Patient Education by Pharmacist Programme	Usual care	x	x	x	x						
(Cani et al., 2015), Brazil	Parallel RCT, 6	No. of Patients (IG/CG): 37/41 Loss to follow-up (IG/CG): 3/5 Age (IG/CG): 61.91 (9.58) / 61.58 (8.14) Gender (IG/CG): 61.7/61.1 Duration of T2DM (IG/CG):14.56 (7.40)/14.92 (8.49)	Individualized pharmacotherapeutic care plan	Standard care	x				x		x	x		
(Castejón et al., 2014), USA	Parallel RCT, 5	No. of Patients (IG/CG): 19/24 Loss to follow-up (IG/CG): not mentioned Age (IG/CG): 54 (9)/55 (10) Gender (IG/CG):36.8/20.8 Duration of T2DM (IG/CG): not mentioned	Pharmacist counseling sessions	Usual care		x								
(Chan et al., 2012), Hong Kong	Parallel RCT, 9	No. of Patients (IG/CG): 51/54 Loss to follow-up (IG/CG): 0/0 Age (IG/CG): 63.2 (9.5)/ 61.7 (11.2) Gender (IG/CG) female (%): 41.2/48.1 Duration of T2DM (IG/CG):14.9 (5.6)/13.8 (6.8)	Pharmacist Care Program	Routine medical care	x		x	x						
(Chen et al., 2016), Taiwan	Parallel RCT, 6	No. of Patients (IG/CG): 50/50 Loss to follow-up (IG/CG): 0/0 Age (IG/CG):72.16 (6.6)/72.76 (5.9) Gender (IG/CG): 50/50 Duration of T2DM (IG/CG): not mentioned	Pharmaceutical care	Usual care	x		x		x					
(Hayward et al., 2005), USA	Parallel RCT, 24	No. of Patients (IG/CG): 41/39 Loss to follow-up (IG/CG): 5/10 Age (IG/CG): 52.2 ± 11.2/51.0 ± 9.0 Gender (IG/CG) female (%): 41.2/43.9 Duration of T2DM (IG/CG): not mentioned	Clinical pharmacist led diabetes self-management education	Usual care		x	x							
(Chow et al., 2015), Malaysia	Parallel RCT, 6	No. of Patients (IG/CG): 75/75 Loss to follow-up (IG/CG): 25/7 Age (IG/CG): not mentioned Gender (IG/CG): 64/62.7 Duration of T2DM (IG/CG):8.3 ± 4.10/8.90 ± 6.00	Pharmacist-led patient education	Usual care	x	x	x	x						
(Chung et al., 2014), Malaysia	Parallel RCT, 12	No. of Patients (IG/CG): 120/121 Loss to follow-up (IG/CG): Not mentioned Age (IG/CG): 59.7 (9.5)/ 58.5 (8.3) Gender (IG/CG) female (%): 58.3/53.7 Duration of T2DM (IG/CG):16.3 (8)/16.3 (8)	Pharmaceutical care model	Standard primary care			x	x	x			x		
(Clifford et al., 2005), Australia	Parallel RCT, 12	No. of Patients (IG/CG): 99/99 Loss to follow-up (IG/CG): 7/11 Age (IG/CG): 70.5 (7.1)/70.3 (8.3) Gender (IG/CG) female (%): 42.2/43.2 Duration of T2DM (IG/CG):10.0 /8.0	Pharmaceutical care program	Usual care		x	x	x	x				x	
(Cohen et al., 2011), USA	Parallel RCT, 6	No. of Patients (IG/CG): 53/50 Loss to follow-up (IG/CG): 5/2 Age (IG/CG):69.8 (10.7)/67.2 (9.4) Gender (IG/CG) female (%): 0/4 Duration of T2DM (IG/CG): Not mentioned	Pharmacist-led group medical visit program	Standard primary care	x	x		x	x			x		
(Doucette et al., 2009), USA	Parallel RCT, 12	No. of Patients (IG/CG): 36/42 Loss to follow-up (IG/CG): 5/7 Age (IG/CG): 58.7 (13.3)/ 61.2 (10.9) Gender (IG/CG): 61.8/53.7 Duration of T2DM (IG/CG): not mentioned	Pharmaceutical Care	Usual care		x	x	x	x					x
(Farsaei et al., 2011), Iran	Parallel RCT, 3	No. of Patients (IG/CG): 87/87 Loss to follow-up (IG/CG): Not mentioned Age (IG/CG):53.4 (9.8)/52.9 (8.5) Gender (IG/CG) female (%):63.2/68.2 Duration of T2DM (IG/CG):10.8 (5.3)/10.3 (8.2)	Clinical pharmacist-led patient education program	General education offered by the nursing staff		x	x	x					x	
(Fornos et al., 2006), Spain	Parallel RCT, 13	No. of Patients (IG/CG): 58/56 Loss to follow-up (IG/CG): 2/0 Age (IG/CG): 62.4 (10.5)/ 64.9 (10.9) Gender (IG/CG) female (%): 57.1/57.1 Duration of T2DM (IG/CG): not mentioned	Pharmacotherapy Program	Usual care			x		x			x		
(Jameson and Baty, 2010), USA	Parallel RCT, 12	No. of Patients (IG/CG): 52/51 Loss to follow-up (IG/CG): not mentioned Age (IG/CG): 49.3 (10.8)/49.7 (10.9) Gender (IG/CG): 51.1/51 Duration of T2DM (IG/CG): not mentioned	Pharmacist management of diabetes	Usual care		x	x		x					x
(Jacobs et al., 2012), USA	Parallel RCT, 12	No. of Patients (IG/CG): 195/201 Loss to follow-up (IG/CG): 22/24 Age (IG/CG): 62.7 ± 10.8/63.0 ± 11.2 Gender (IG/CG) female (%): 32/45 Duration of T2DM (IG/CG): not mentioned	Pharmacist Assisted Medication Program Enhancing the Regulation of Diabetes	Usual care	x	x			x				x	
(Jahangard-Rafsanjani et al., 2015), Iran	Parallel RCT, 6	No. of Patients (IG/CG): 51/50 Loss to follow-up (IG/CG): 6/10 Age (IG/CG): 57.3 (8.6)/ 55.9 (8.7) Female Gender (IG/CG): 49/52 Duration of T2DM (IG/CG):4.6 (4.3)/5.7 (5.9)	Diabetes Education Program	Usual care	x	x		x		x	x		x	x
(Jarab et al., 2012), Jordan	Parallel RCT, 6	No. of Patients (IG/CG): 85/86 Loss to follow-up (IG/CG): 8/7 Age (IG/CG): 63.4 [10.1]/65.3 [9.2] Gender (IG/CG) female (%): 36/38 Duration of T2DM (IG/CG):9.7 (7.4)/10.1 (7.7)	Comprehensive clinical pharmacy service	Usual care	x	x	x	x	x	x		x		
(Kang et al., 2010), Taiwan	Parallel RCT,	No. of Patients (IG/CG): 33/34 Loss to follow-up (IG/CG): 5/6 (IG/CG): 55.3 (7.7)/ 51.7 (8.5) Gender (IG/CG): 42.8/50 Duration of T2DM (IG/CG):3.8 (3.2)/4.4 (3.0)	Family partnership intervention care (FPIC)	Conventional care		x	x					x	x	
(Ko et al., 2007), South Korea	Parallel RCT, 48	No. of Patients (IG/CG): 219/218 Loss to follow-up (IG/CG): 49/70 Age (IG/CG):53.3 ± 9.3/54.1 ± 7.4 Gender (IG/CG): 58/54.2 Duration of T2DM (IG/CG): 6.0 ± 6.0/6.2 ± 5.5	Structured intensive diabetes education programme	Standard care	x	x	x					x		x
(Korcegez et al., 2017), USA	Parallel RCT, 12	No. of Patients (IG/CG): 79/80 Loss to follow-up (IG/CG): 4/3 Age (IG/CG): 61.80 ± 10.38/ 62.22 ± 9.54 Gender (IG/CG) female (%):: 77.3/74 Duration of T2DM (IG/CG): not mentioned	Pharmacist-Led Program	Usual care	x	x	x		x	x		x		x
(Krass et al., 2007), Australia	Cluster RCT, 6	No. of Patients (IG/CG): 176/159 Loss to follow-up (IG/CG): 33/39 Age (IG/CG): 62 (11)/62 (11) Gender (IG/CG) female (%): 49/49 Duration of T2DM (IG/CG): not mentioned	Community pharmacy diabetes service model	Usual care	x			x						x
(Lim et al., 2016), Malaysia	Parallel RCT, 12	No. of Patients (IG/CG): 50/50 Loss to follow-up (IG/CG): 11/13 Age (IG/CG): 55.62 (1.49)/ 57.00 (1.56) Gender (IG/CG): 53.8/54.1 Duration of T2DM (IG/CG): not mentioned	Diabetes Medication Therapy Adherence Clinic	Usual care	x		x	x		x		x		
Mahwi and Obied 2013, Iraq	Parallel RCT, 4	No. of Patients (IG/CG): 65/65 Loss to follow-up (IG/CG): Age (IG/CG): 52 ± 7.86/53.4 ± 10.81 Gender (IG/CG) female (%): 71/67.2 Duration of T2DM (IG/CG):4.12± 3.42/5.09± 4.42	Pharmaceutical care program	Traditional medical care			x	x	x					
(Mehuys et al., 2011), Belgium	Cluster RCT, 24	No. of Patients (IG/CG): 153/135 Loss to follow-up (IG/CG): 5/3 Age (IG/CG): 63.0 (40–84)/ 62.3 (45–79) Gender (IG/CG) female (%): 49/ 46.3 Duration of T2DM (IG/CG): not mentioned	Standard diabetes education program	Usual pharmacist care	x		x	x				x		
(Mourão et al., 2013), Brazil	Parallel RCT, 6	No. of Patients (IG/CG): 65/64 Loss to follow-up (IG/CG): 25/24 Age (IG/CG): 60.0 (10.2)/61.3 (9.9) Gender (IG/CG) female (%): 68/66 Duration of T2DM (IG/CG): not mentioned	Pharmaceutical care program	Usual health care	x	x	x	x	x			x		x
(Nascimentoa et al., 2016), Spain	Parallel RCT, 6	No. of Patients (IG/CG): 44/43 Loss to follow-up (IG/CG): 0/0 Age (IG/CG): 74.2 (5.4)/72.3 (4.5) Gender (IG/CG): 43.2/41.9 Duration of T2DM (IG/CG): 10.4 (6.9) / 14.7 (8.5)	Individualized pharmacotherapy management service		x				x					
(Odegard et al., 2005), USA	Parallel RCT, 12	No. of Patients (IG/CG): 43/34 Loss to follow-up (IG/CG): 4/7 Age (IG/CG):51.6 (11.6)/51.9 (10.4) Gender (IG/CG) female (%): 48/38 Duration of T2DM (IG/CG):6.9 (5.3)/8.3 (7.5)	Diabetes Care Plan	Usual care			x	x	x					
(Rothman et al., 2005), USA	Parallel RCT, 12	No. of Patients (IG/CG): 112/105 Loss to follow-up (IG/CG): 13/10 Age (IG/CG): 46.1 /42.3 Gender (IG/CG) female (%): 56/56 Duration of T2DM (IG/CG):8 (9)/9 (9)	Pharmacist-led, primary care–based, disease management program	Usual care			x	x	x					
(Samtia et al., 2013), Pakistan	Parallel RCT, 5	Age (IG/CG): 46.1 (23-74)/ 42.3 (21–77) Loss to follow-up (IG/CG): 4/2 Age (IG/CG): 54 (13) /57 (11) Gender (IG/CG): 47.2/51.2 Duration of T2DM (IG/CG): not mentioned	Multifactorial Intervention		x	x		x				x		x
(Sarkadi and Rosenqvist, 2004), Sweden	Parallel RCT, 24	No. of Patients (IG/CG): 39/38 Loss to follow-up (IG/CG): 6/7 Age (IG/CG):66.4 (7.9)/66.5 (10.7) Gender (IG/CG) female (%): not mentioned Duration of T2DM (IG/CG):5.9 (5.8)/2.6 (2.2)	Pharmacist-led educational program	Usual care	x	x				x		x		x
Scott et al. 2006, USA	Parallel RCT, 9	No. of Patients (IG/CG): 76/73 Loss to follow-up (IG/CG): 12/6 Age (IG/CG): not mentioned Gender (IG/CG): 57.9/64.4 Duration of T2DM (IG/CG): not mentioned	Pharmacist-managed diabetes care services	Usual care					x			x		
(Shao et al., 2017), China	Parallel RCT, 6	No. of Patients (IG/CG): 120/120 Loss to follow-up (IG/CG): 20/21 Age (IG/CG): 58.86 ± 10.59/59.20 ± 10.34 Gender (IG/CG): 49/42.5 Duration of T2DM (IG/CG): not mentioned	Pharmaceutical care	Usual care	x	x	x					x		x
(Siaw et al., 2017), Singapore	Parallel RCT, 6	No. of Patients (IG/CG): 214/197 Loss to follow-up (IG/CG): Age (IG/CG):59.2 ± 8.2/60.1 ± 8.1 Gender (IG/CG): 47.7/39.1 Duration of T2DM (IG/CG):12.7 ± 9.1/13.5 ± 8.9	Multidisciplinary collaborative care	Usual care -physician-centered care	x	x								
(Sriram et al., 2011), India	Parallel RCT, 8	No. of Patients (IG/CG): 60/60 Loss to follow-up (IG/CG): not mentioned Age (IG/CG): 53.65 (2.38) / 57.98 (2.62) Gender (IG/CG) female (%): 50/50 Duration of T2DM (IG/CG): not mentioned	Pharmaceutical care	Usual care	x	x				x		x	x	
(Suppapitiporn et al., 2005), Thailand	Parallel RCT, 6	No. of Patients (IG/CG): 180/180 Loss to follow-up (IG/CG): not mentioned Age (IG/CG): 61.4 (10.6)/59.9 (11.5) Gender (IG/CG) female (%): 67.2/64.4 Duration of T2DM (IG/CG): not mentioned	Disease counseling and education + diabetic information booklet + special medication container	Usual care	x		x	x		x	x		x	x
(Taveira et al., 2010), USA	Parallel RCT, 6	No. of Patients (IG/CG): 64/54 Loss to follow-up (IG/CG): 6/3 Age (IG/CG):62.2 (10.3)/66.8 (10.2) Gender (IG/CG) female (%): 8.6/0 Duration of T2DM (IG/CG): not mentioned	Pharmacist-led group medical visit program	Usual care			x		x					
(Taylor et al., 2005), Australia	Parallel RCT, 9	No. of Patients (IG/CG): 53/46 Loss to follow-up (IG/CG): Age (IG/CG):65 / 66 Gender (IG/CG): 54.7/56.5 Duration of T2DM (IG/CG): not mentioned	Specialized service	Usual care	x	x		x	x			x		x
(Tourkmani et al., 2016), Saudi Arabia	Parallel RCT, 9	No. of Patients (IG/CG): 140/122 Loss to follow-up (IG/CG): 73/8 Age (IG/CG):55.12 (12.76)/56.06 (11.08) Gender (IG/CG): 60/66.4 Duration of T2DM (IG/CG): not mentioned	Ramadan focused education program	Standard diabetic care	x	x				x		x		x
(Wishah et al., 2015), Jordan	Parallel RCT, 6	No. of Patients (IG/CG): 52/54 Loss to follow-up (IG/CG): 2/3 Age (IG/CG): 52.9 (9.6)/53.2 (11.2) Gender (IG/CG) female (%): 61.5/51.9 Duration of T2DM (IG/CG):5.5 (4.5)/5.1 (4.9)	Pharmaceutical care interventions developed by the clinical pharmacist	Usual care provided by the medical and nursing staff	x	x	x	x	x	x				

*IG, Intervention group; CG, control group; T2DM, type 2 diabetes mellitus; SD, Standard deviation*.

### Risk of bias

**Figure 2:**
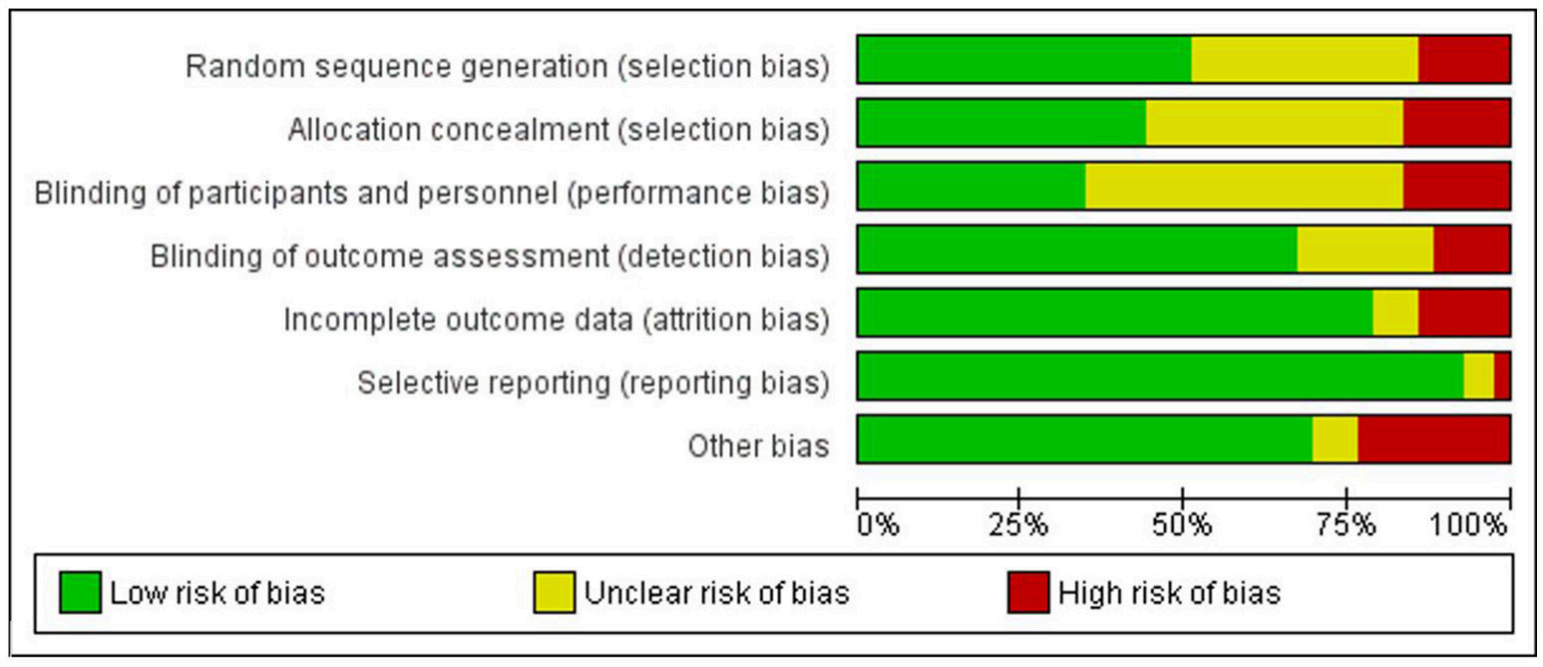
Overall risk of bias graph.

**Figure 3:**
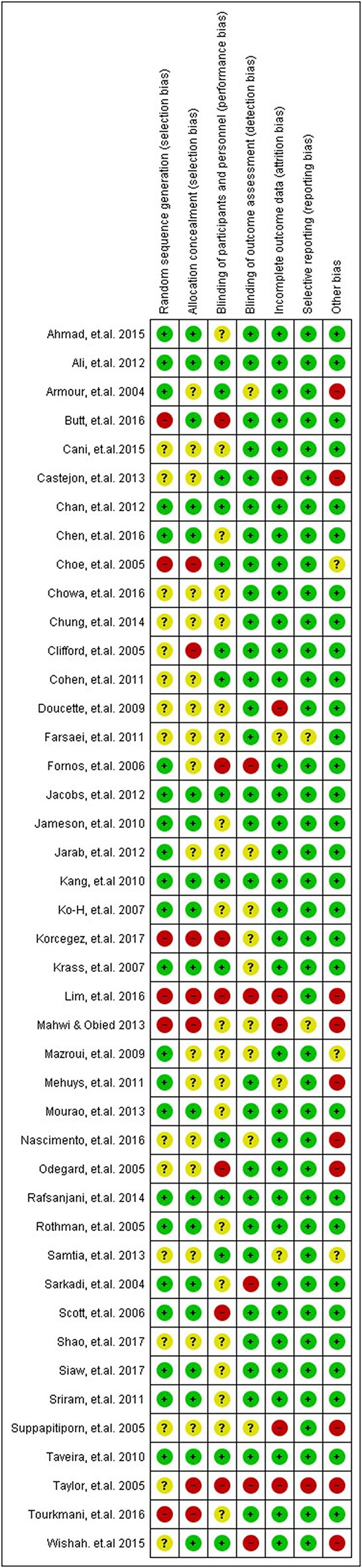
Summary of ROB assessment of the included studies.

The ROB for included RCTs (*n* = 43 studies) using Cochrane ROB tool is presented in Figure 2 (ROB graph) and Figure 3 (ROB summary). More than 75% of the studies were free of attrition bias, reporting bias and other sources of bias. Performance bias and selection bias, were granted in only 30 and 40% of the studies, respectively.

### Primary outcome

#### Glycosylated hemoglobin (HbA1c)

HbA1c was the primary clinical outcome for all of the included studies (*n* = 43). Pair-wise meta-analysis (Supplementary Figure 1) shows an overall effect in favor of the pharmacist based educational interventions (irrespective of nature of intervention) on HbA1c, where the levels of HbA1c reduced with a mean difference of −0.85% [95% CI −0.96, −0.75; *p* < 0.001]. But there was a substantial heterogeneity (*I*^2^ = 78.8%). Sub-group analysis revealed that larger effect was made by studies which involved pharmacist based diabetes education [−0.90%; 95% CI −1.07, −0.74; *p* < 0.001; *I*^2^ = 87%], followed by studies which involved pharmacist based diabetes education plus pharmaceutical care [−0.83; 95% CI −0.98, −0.67; *p* < 0.001; *I*^2^ = 68%], and studies in which diabetes education was provided by health care team involving pharmacist [−0.72; 95% CI −1.02, −0.43; *p* < 0.188; *I*^2^ = 37.3%]. Heterogeneity remained substantially high after performing the subgroup analysis (Supplementary Figure 2). After discussion and mutual consensus *n* = 4 studies (Odegard et al., 2005; Al Mazroui et al., 2009; Farsaei et al., 2011; Mehuys et al., 2011) which were showing selection bias, detection bias and other sources of bias, and were significantly contributing toward heterogeneity were excluded from the analysis. After removing these studies the heterogeneity reduced substantially from 87 to 43.3% and from 68 to 62.9% in the sub-groups which involved pharmacist based diabetes education and pharmacist based diabetes education plus pharmaceutical care, respectively.

Final sub-group analysis revealed that studies which examined pharmacist based diabetes education plus pharmaceutical care interventions showed comparatively similar effect in terms of reduction in HbA1c levels [−0.86; 95% CI −1.01, −0.71; *p* < 0.001; *I*^2^ = 62.9%], as shown by studies which involved diabetes education by pharmacist [−0.85; 95% CI −0.95, −0.75; *p* < 0.04; *I*^2^ = 43.4%] and those studies which involved diabetes education by health care team which included pharmacist as part of team [−0.72; 95% CI −1.02, −0.43; *p* < 0.188; *I*^2^ = 37.3%], details are presented in Supplementary Figure 3.

#### Moderation of effect for study features on glycosylated hemoglobin

The results of subgroup analysis for studies on the basis of geographical area revealed that the studies which were conducted in Asia (*n* = 20), had greater reductions in HbA1c levels (−1.02; 95%CI −1.16, −0.88; *p* < 0.001] as compared the studies which were conducted in other subcontinents (details are presented in Supplementary Table 3). No significant difference on mean difference of HbA1c was observed when other features of the studies, such as baseline HbA1c levels and duration of invention, were examined (Supplementary Table 3). Similarly the four studies (Odegard et al., 2005; Al Mazroui et al., 2009; Farsaei et al., 2011; Mehuys et al., 2011) which were showing selection bias and detection bias, and were also contributing toward significant heterogeneity, were not significant effect modifier, details shown in Supplementary Table 3.

#### Network meta-analysis

**Figure 4:**
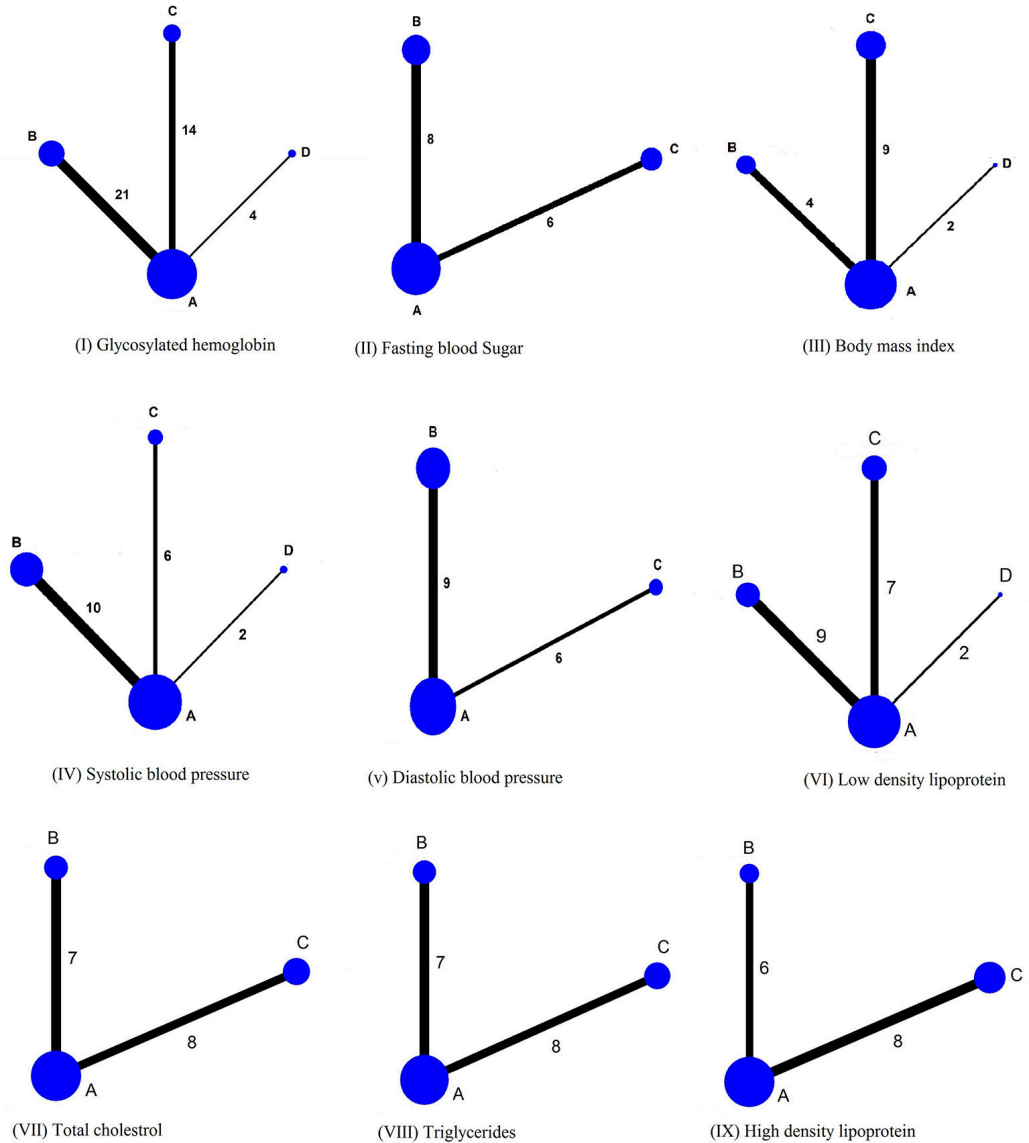
Network plot. The width of lines for each connection in the evidence network is proportional to the number of randomized controlled studies that compared each point of treatment. The sizes of the nodes are proportional to the number of patients. A, Usual care; B, Pharmacist based diabetes education plus pharmaceutical care; C, Pharmacist based diabetes education; D, Diabetes education by health care team involving pharmacist as member.

Thirty nine studies (5534 participants with type 2 diabetes) were finally included in the NMA. Evidence network for clinical outcomes examined in current study is shown in Figure 4.

When reference arm was set as usual care in the analysis, all interventions, irrespective of the nature, reduced HbA1c significantly as compared to usual care, and none of the intervention was statistically better from the other. Surface under cumulative ranking cure plot (SUCRA) (Supplementary Figure 24) showed that the interventions with pharmacist based diabetes education plus pharmaceutical care was the best intervention [−0.86, 95% CI −0.983, −0.727; Z −13.07; *p* < 0.001], followed by interventions which delivered diabetes education by pharmacist [−0.83; 95% CI −0.975, −0.686; *p* < 0.001; Z −11.26], and diabetes education by health care team involving pharmacist as member [−0.72; 95%CI −1.036, −0.414; *p* < 0.001; Z −4.57], as shown in Table 2.

**Table 2:** Network meta-analysis for impact various pharmacist based interventions on primary and secondary clinical outcomes of type 2 diabetes patients in comparison to usual care.

**Outcome parameter**	**Intervention**	**MD [95% CI]**	**SE**	***Z***	***I*^2^**	***p*-value**
HbA1c	Pharmacist based Diabetes education plus Pharmaceutical care	−0.86 [−0.983, −0.727]	0.0654	−13.07	55.89%	<0.001
	Pharmacist based Diabetes education	−0.83 [−0.975, −0·686]	0.0737	−11.26		<0.001
	Diabetes education by Health care team involving pharmacist as member	−0.72 [−1.036, −0.414]	0.1586	−4.57		<0.001
FBS (mg/dL)	Pharmacist based Diabetes education plus Pharmaceutical care	−31.89 [−42.307, −21.481]	5.313	−6.00	63.43%	<0.001
	Pharmacist based Diabetes education	−25.88 [−36.06, −15.71]	5.191	−4.99		<0.001
BMI (kg/m^2^)	Pharmacist based Diabetes education plus Pharmaceutical care	−0.56 [−1.008, −0.0114]	0.228	−2.46		0.014
	Pharmacist based Diabetes education	−0.61 [−0.898, −0.321]	0.147	−4.14	52.71%	<0.001
	Diabetes education by Health care team involving pharmacist as member	0.00 [−0.516, 0.525]	0.266	0.02		0.987
SBP (mm Hg)	Pharmacist based Diabetes education plus Pharmaceutical care	−8.12 [−8.46, −3.86]	1.238	−6.55		<0.001
	Pharmacist based Diabetes education	−3.18 [−5.975, −0.377]	1.428	−2.22	53.46%	0.026
	Diabetes education by Health care team involving pharmacist as member	−4.34 [−9.300, 0.619]	2.530	−1.72		0.086
DBP (mm Hg)	Pharmacist based Diabetes education plus Pharmaceutical care	−3.19 [−4.792, −1.579]	0.820	−3.89	58.52%	<0.001
	Pharmacist based Diabetes education	−1.63 [−3.215, −0.042]	0.810	−2.01		0.044
LDL (mmol/L)	Pharmacist based Diabetes education plus Pharmaceutical care	−0.36 [−0.503, −0.225]	0·071	−5·14		<0.001
	Pharmacist based Diabetes education	−0.35 [−0.502, −0.225]	0.078	−4.44	55.68%	<0.001
	Diabetes education by Health care team involving pharmacist as member	−0.06 [−0.382, 0.270]	0.166	−0.34		0.735
TG (mmol/L)	Pharmacist based Diabetes education plus Pharmaceutical care	−0.41 [−0.601, −0.220]	0.097	−4.23	55.71%	<0.001
	Pharmacist based Diabetes education	−0.15 [−0.317, −0.014]	0.084	−1.8		0.073
HDL (mmol/L)	Pharmacist based Diabetes education plus Pharmaceutical care	0.10 [−0.004, 0.196]	0.051	1.88	84.71%	0.061
	Pharmacist based Diabetes education	0.01 [−0.071, 0·098]	0.043	0.31		0.755
TC (mmol/L)	Pharmacist based Diabetes education plus Pharmaceutical care	−0.17 [−0.447, 0·110]	0.142	−1.19	78.19%	0.236
	Pharmacist based Diabetes education	−0.27 [−0.542, 0·004]	0.139	−1.93		0.054

*MD, mean difference; CI, confidence interval; SE, standard error; HbA1c, glycosylated hemoglobin; FBS, fasting blood sugar; BMI, body mass index; SBP, systolic blood pressure; DBP, diastolic blood pressure; LDL, low density lipoprotein; TG, triglycerides; HDL, high density lipoprotein; TC, total cholesterol*.

**Figure 5:**
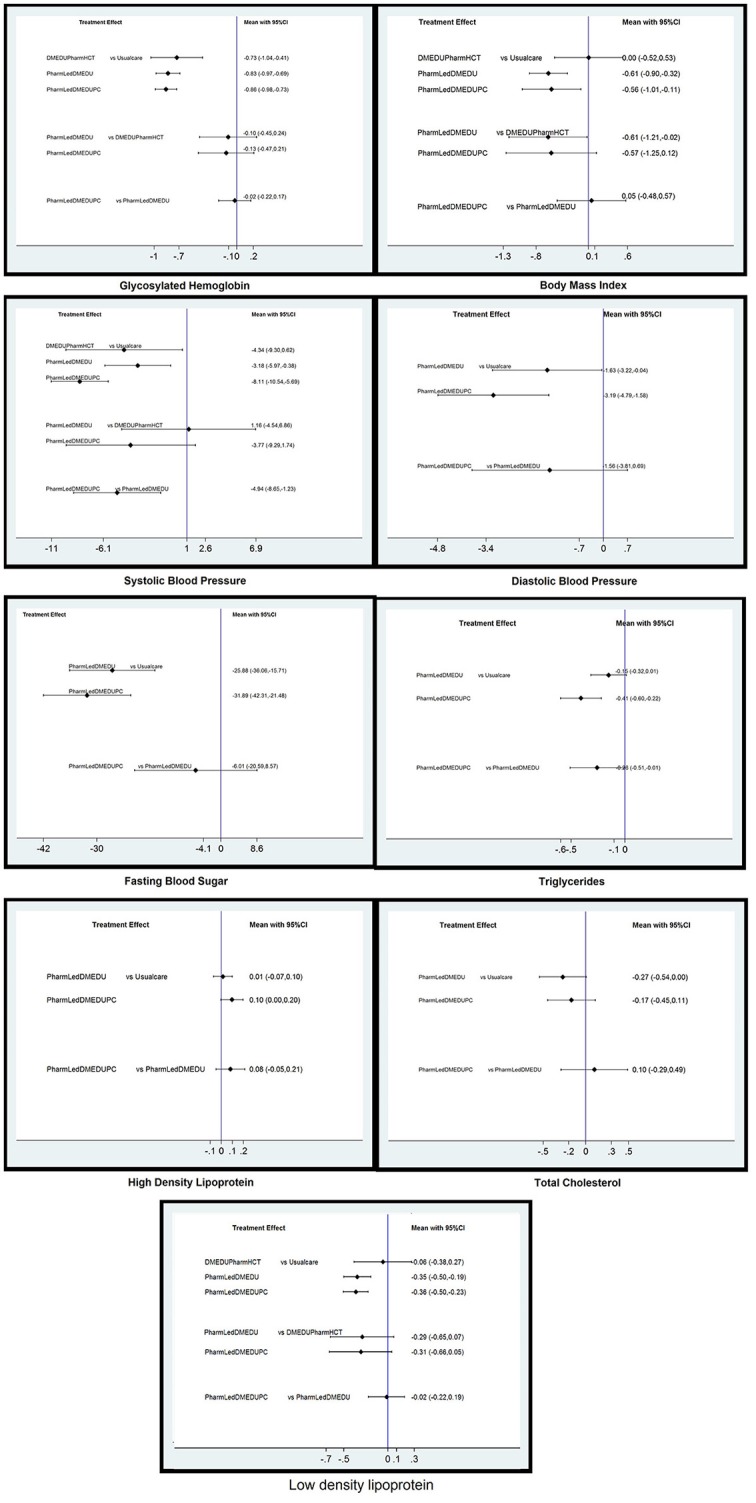
Network meta-analysis estimates of changes in primary and secondary clinical outcomes of type 2 diabetes patients. PharmLedDMEDUPC, Pharmacist based diabetes education plus pharmaceutical care; PharmLedDMEDU, Pharmacist based diabetes education; DMEDUPharmHCT, Diabetes education by health care team involving pharmacist as member.

League table was generated by using NMA to present all possible pairwise comparisons between any two of the three interventions and traditional pairwise meta-analysis (Table 3). It was evident from the NMA that all of three interventions show a comparable efficacy in lowering HbA1c levels (Table 3). The Pharmacist based diabetes education plus pharmaceutical care was not found statistically significant in lowering the HbA1c levels of type 2 diabetes patients as compared to the interventions which involved diabetes education by pharmacist without involving pharmaceutical care [−0.02%; 95% CI −0.22, 0.17] and diabetes education by health care team involving pharmacist as member of team [−0.13%; 95% CI −0.45, 0.24], details are presented in Figure 5.

**Table 3:** Network meta-analysis and pairwise meta-analysis of various pharmacy based interventions on primary and secondary clinical outcomes.

**INTERVENTIONS EFFECT ON HbA1c (%)**			
Pharm-based DM EDU + PC	ND	ND	**−0.83 [−0.98, −0.67]**
−0.02 [−0.22, 0.17]	Pharm-Based DM EDU	ND	**−0.90 [−1.·07, −0.74]**
−0.13 [−0.47, 0.21]	−0.10 [−0.45, 0.24]	DM EDU Pharm + HCT	**−0.72 [−1.02, −0.43]**
**−0.86 [−0.983, −0.727]**	**−0.83 [−0.975, −0.686]**	**−0.73 [−1.036, −0.414]**	Usual care
**INTERVENTIONS EFFECT ON BODY MASS INDEX (kg/m^2^)**			
Pharm-based DM EDU + PC	ND	ND	**−0.84 [−1.47, −0.20]**
0·05 [−0.48, 0.57]	Pharm-Based DM EDU	ND	**−0.50 [−0.90, −0.11]**
**−0.57 [−1.25, −0.12]**	**−0.61 [−1.21, −0.02]**	DM EDU Pharm + HCT	−0.02 [−0.31, 0.28]
**−0.56 [−1.01, −0.11]**	**−0.61 [−0.90, −0.32]**	0.00 [−0.52, 0.53]	Usual care
**INTERVENTIONS EFFECT ON SYSTOLIC BLOOD PRESSURE (mm Hg)**			
Pharm-based DM EDU + PC	ND	ND	**–8.18 [−10.97, −5.39]**
**−4.94 [−8.65, −1.23]**	Pharm-Based DM EDU	ND	−2.16 [−5.04, 0.71]
−3.77 [−9.29, 1.74]	1.16 [−4.54, 6.86]	DM EDU Pharm + HCT	**−4.06 [−6.94, −1.19]**
**−8·11 [−10·54, −5·69]**	**−3·18 [−5·97, −0·38]**	−4·34 [−9·30, 0·62]	Usual care
**INTERVENTIONS EFFECT ON DIASTOLIC BLOOD PRESSURE (mm Hg)**			
Pharm-based DM EDU + PC	ND	**−3.52 [−5.28, −0.72]**	
−1.56 [−3.81, 0.69]	Pharm-Based DM EDU	**−2.66 [−4.61, −0.72]**	
**−3.19 [−4.79, −1.58]**	**−1.63 [−3.22, −0.04]**	Usual care	
**INTERVENTIONS EFFECT ON FASTING BLOOD SUGAR (mg/dL)**			
Pharm-based DM EDU + PC	ND	**−32.06 [−43.47, −20.65]**	
−6.01 [−20.59, 8.57]	Pharm-Based DM EDU	**−36.67 [−52.44, −20.90]**	
**−31.89 [−42.31, −21.48]**	**−25.88 [−36.06, −15.71]**	Usual care	
**INTERVENTIONS EFFECT ON TRIGLYCERIDES (mmol/L)**			
Pharm-based DM EDU + PC	ND	**−0.41 [−0.61, −0.20]**	
**−0.26 [−0.51, −0.01]**	Pharm-Based DM EDU	**−0.15 [−0.31, −0.00]**	
**−0.41 [−0·60, −0.22]**	−0.15 [−0.32, 0.01]	Usual care	
**INTERVENTIONS EFFECT ON HIGH DENSITY LIPOPROTEIN (mmol/L)**			
Pharm-based DM EDU + PC	ND	**0.08 [0.02, 0.15]**	
0.08 [−0.05, 0.20]	Pharm-Based DM EDU	0.01 [−0.06, 0.09]	
**0·10 [0·00, 0·20]**	0·01 [−0·07, 0·10]	Usual care	
**INTERVENTIONS EFFECT ON TOTAL CHOLESTEROL (mmol/L)**			
Pharm-based DM EDU + PC	ND	−0.16 [−0.41, 0·09]	
0·10 [−0·29, 0·49]	Pharm-Based DM EDU	**−0.27 [−0.47, −0.07]**	
−0.17 [−0.45, 0.11]	−0.27 [−0.54, 0.00]	Usual care	
**INTERVENTIONS EFFECT ON LOW DENSITY LIPOPROTEIN (mmol/L)**			
Pharm-based DM EDU + PC	ND	ND	**−0.36 [−0.51, −0.21]**
−0.02 [−0.22, 0.19]	Pharm-Based DM EDU	ND	**−0.35 [−0.49, −0.22]**
−0.31 [−0.66, 0.05]	−0.29 [−0.65, 0.07]	DM EDU Pharm + HCT	−0.06 [−0.30, 0.17]
**−0.36 [−0.50,−0.23]**	**−0·35 [−0.50,−0.19]**	−0.06 [−0.38, 0.27]	Usual care

*In upper right triangle, the results of interventions' effect are presented as mean difference [95%CI], based on traditional pairwise meta-analysis. Whereas, the results of network meta-analysis are shown in lower-left triangle.Pharm-Led DM EDU +PC, Pharmacist based diabetes education plus Pharmaceutical care; Pharm-Led DM EDU, Pharmacist based diabetes education; DM EDU Pharm + HCT, Diabetes education by health care team involving pharmacist as member; MD, mean difference; ND, no data. Lower left results compare row-defining intervention against column-defining intervention. Upper right results compare column-defining intervention against row-defining interventions, where MD < 0 favors column and row defining treatments (Except for high density lipoprotein). All significant results are presented in bold*.

#### Secondary clinical outcomes

Body mass index (BMI), FBS, blood pressure control (systolic and diastolic blood pressure), and, lipid profile (LDL, HDL, triglycerides, and total cholesterol) were the secondary clinical outcome of this review. After final assessment *n* = 20 studies which reported for BMI (Clifford et al., 2005; Fornos et al., 2006; Scott et al., 2006; Krass et al., 2007; Al Mazroui et al., 2009; Kang et al., 2010; Taveira et al., 2010; Ali et al., 2012; Chan et al., 2012; Jarab et al., 2012; Mourão et al., 2013; Samtia et al., 2013; Castejón et al., 2014; Jahangard-Rafsanjani et al., 2015; Wishah et al., 2015; Butt et al., 2016; Lim et al., 2016; Tourkmani et al., 2016; Korcegez et al., 2017; Shao et al., 2017) *n* = 16 for FBS (Clifford et al., 2005; Suppapitiporn et al., 2005; Fornos et al., 2006; Al Mazroui et al., 2009; Farsaei et al., 2011; Sriram et al., 2011; Jarab et al., 2012; Mahwi and Obied, 2013; Mourão et al., 2013; Samtia et al., 2013; Chung et al., 2014; Wishah et al., 2015; Butt et al., 2016; Lim et al., 2016; Korcegez et al., 2017; Shao et al., 2017) *n* = 19 studies for SBP (Clifford et al., 2005; Fornos et al., 2006; Scott et al., 2006; Al Mazroui et al., 2009; Doucette et al., 2009; Taveira et al., 2010; Cohen et al., 2011; Ali et al., 2012; Chan et al., 2012; Jacobs et al., 2012; Mourão et al., 2013; Castejón et al., 2014; Ahmad et al., 2015; Jahangard-Rafsanjani et al., 2015; Lim et al., 2016; Tourkmani et al., 2016; Korcegez et al., 2017; Shao et al., 2017; Siaw et al., 2017) *n* = 17 studies for DBP (Clifford et al., 2005; Fornos et al., 2006; Scott et al., 2006; Al Mazroui et al., 2009; Doucette et al., 2009; Correr et al., 2011; Ali et al., 2012; Chan et al., 2012; Jacobs et al., 2012; Jarab et al., 2012; Mourão et al., 2013; Castejón et al., 2014; Ahmad et al., 2015; Jahangard-Rafsanjani et al., 2015; Lim et al., 2016; Tourkmani et al., 2016; Korcegez et al., 2017; Shao et al., 2017) *n* = 18 studies for lipid profile (Fornos et al., 2006; Al Mazroui et al., 2009; Doucette et al., 2009; Kang et al., 2010; Taveira et al., 2010; Cohen et al., 2011; Ali et al., 2012; Chan et al., 2012; Jacobs et al., 2012; Jarab et al., 2012; Mourão et al., 2013; Castejón et al., 2014; Wishah et al., 2015; Butt et al., 2016; Lim et al., 2016; Tourkmani et al., 2016; Korcegez et al., 2017; Shao et al., 2017) were included for meta-analysis. Mean difference was estimated for all the studies included in analysis.

The meta-analysis showed an overall effect in favor of the interventions on BMI [−0.54; 95%CI −0.85, −0.23; *p* < 0.001, *I*^2^ = 87.3%] (Supplementary Figure 4), FBS [−34.95; 95%CI −46.22, −23.69; *p* < 0.001; *I*^2^ = 93.5%] (Supplementary Figure 7), SBP [−5.40; 95%CI −7.56, −3.24; *p* < 0.001; *I*^2^ = 78.9%] (Supplementary Figure 10), DBP [−3.12; 95%CI −4.41, −1.84; *p* < 0.001; *I*^2^ = 80.6%] (Supplementary Figure 13), LDL [−0.33; 95%CI −0.43, −0.23; *p* = 0.001; *I*^2^ = 58.3%] (Supplementary Figure 16), triglycerides [−0.26; 95%CI −0.39, −0.12; *p* < 0.001; *I*^2^ = 64.3%] (Supplementary Figure 18), total cholesterol [−0.21; 95%CI −0.35, −0.07; *p* < 0.001; *I*^2^ = 74.8%] (Supplementary Figure 20). Whereas, on HDL no favorable effect was observed [0.04; 95%CI −0.02, 0.09; *p* = 0.18; *I*^2^ = 84.6%] (Supplementary Figure 22).

Sub-group analysis were performed for the secondary clinical outcomes, in order to examine the comparative efficacy of different pharmacist based interventions. However, heterogeneity was not significantly reduced in the sub-group analysis for some of the secondary clinical outcomes (as shown Supplementary Figures 5, 8, 11, 14).

After discussion and mutual consensus the studies with performance bias, selection bias, and, which were contributing to significant heterogeneity were excluded from the final subgroup analysis. Therefore, *n* = 5 studies for BMI (Al Mazroui et al., 2009; Ali et al., 2012; Mourão et al., 2013; Tourkmani et al., 2016; Korcegez et al., 2017) *n* = 2 studies for FBS (Sriram et al., 2011; Lim et al., 2016) and one study for systolic blood pressure (Castejón et al., 2014) and *n* = 2 studies for diastolic blood pressure (Al Mazroui et al., 2009; Ahmad et al., 2015) were excluded from analysis. Whereas, *n* = 1 study was not included in the final sub-group analysis, because it was the only study reporting diastolic blood pressure intervention which involved diabetes education by health care professionals involving pharmacist as member (Taveira et al., 2010).

Final sub-group analysis revealed that studies which examined pharmacist based diabetes education plus pharmaceutical care interventions showed maximum reduction in the levels of FBS [−32.06, 95% CI −35.47, −20.65; *p* = 0.014; *I*^2^ = 60.0%] (Supplementary Figure 9), SBP [−8.18; 95% CI −10.97, −5.39; *p* = 0.008; *I*^2^ = 59.6%] (Supplementary Figure 12), DBP [−3.15; 95% CI −5.08, −1.21; *p* = 0.010; *I*^2^ = 60.3%] (Supplementary Figure 15), LDL [−0.36; 95%CI −0.51, −0.21; *p* < 0.00001; *I*^2^ = 63.8%] (Supplementary Figure 17), triglycerides [−0.41; 95%CI −0.61, −0.20; *p* < 0.0001; *I*^2^ = 60.0%] (Supplementary Figure 19), and, HDL [0.08; 95%CI 0.02, 0.15; *p* = 0.04; *I*^2^ = 57.3%] (Supplementary Figure 23). Whereas, diabetes education without involving pharmaceutical care by pharmacist exhibited maximum efficacy on BMI [−0.62; 95%CI −0.92, −0.31, *p* = 0.012] (Supplementary Figure 6), and total cholesterol [−0.27, 95%CI −0.47, −0.07; *p* = 0.0002; *I*^2^ = 75.8%] (Supplementary Figure 21).

### Network meta-analysis

For NMA *n* = 14 studies (2392 type 2 diabetes participants) for FBS (Clifford et al., 2005; Suppapitiporn et al., 2005; Fornos et al., 2006; Al Mazroui et al., 2009; Farsaei et al., 2011; Jarab et al., 2012; Mahwi and Obied, 2013; Mourão et al., 2013; Samtia et al., 2013; Chung et al., 2014; Wishah et al., 2015; Butt et al., 2016; Korcegez et al., 2017; Shao et al., 2017) *n* = 15 studies (2039 type 2 diabetes participants) for BMI (Clifford et al., 2005; Fornos et al., 2006; Scott et al., 2006; Krass et al., 2007; Kang et al., 2010; Taveira et al., 2010; Chan et al., 2012; Jarab et al., 2012; Samtia et al., 2013; Castejón et al., 2014; Jahangard-Rafsanjani et al., 2015; Wishah et al., 2015; Butt et al., 2016; Lim et al., 2016; Shao et al., 2017) *n* = 18 studies (2733 type 2 diabetes participants) for SBP (Clifford et al., 2005; Fornos et al., 2006; Scott et al., 2006; Al Mazroui et al., 2009; Doucette et al., 2009; Taveira et al., 2010; Cohen et al., 2011; Ali et al., 2012; Chan et al., 2012; Jacobs et al., 2012; Mourão et al., 2013; Ahmad et al., 2015; Jahangard-Rafsanjani et al., 2015; Lim et al., 2016; Tourkmani et al., 2016; Korcegez et al., 2017; Shao et al., 2017; Siaw et al., 2017) *n* = 15 studies (1877 type 2 diabetes participants) for diastolic blood pressure (Clifford et al., 2005; Fornos et al., 2006; Scott et al., 2006; Doucette et al., 2009; Ali et al., 2012; Chan et al., 2012; Jacobs et al., 2012; Jarab et al., 2012; Mourão et al., 2013; Castejón et al., 2014; Jahangard-Rafsanjani et al., 2015; Lim et al., 2016; Tourkmani et al., 2016; Korcegez et al., 2017; Shao et al., 2017) *n* = 18 studies (2151 type 2 diabetes participants) for LDL (Fornos et al., 2006; Al Mazroui et al., 2009; Doucette et al., 2009; Kang et al., 2010; Taveira et al., 2010; Cohen et al., 2011; Ali et al., 2012; Chan et al., 2012; Jacobs et al., 2012; Jarab et al., 2012; Mourão et al., 2013; Castejón et al., 2014; Wishah et al., 2015; Butt et al., 2016; Lim et al., 2016; Tourkmani et al., 2016; Korcegez et al., 2017; Shao et al., 2017) *n* = 14 studies (1837 type 2 diabetes participants) for HDL,(Clifford et al., 2005; Fornos et al., 2006; Al Mazroui et al., 2009; Ali et al., 2012; Chan et al., 2012; Jarab et al., 2012; Mourão et al., 2013; Castejón et al., 2014; Wishah et al., 2015; Butt et al., 2016; Lim et al., 2016; Tourkmani et al., 2016; Korcegez et al., 2017; Shao et al., 2017) *n* = 15 studies (2069 type 2 diabetes participants) for triglycerides (Clifford et al., 2005; Fornos et al., 2006; Krass et al., 2007; Al Mazroui et al., 2009; Ali et al., 2012; Chan et al., 2012; Jarab et al., 2012; Mourão et al., 2013; Castejón et al., 2014; Wishah et al., 2015; Butt et al., 2016; Lim et al., 2016; Tourkmani et al., 2016; Korcegez et al., 2017; Shao et al., 2017) and, *n* = 15 studies (2069 type 2 diabetes participants) for total cholesterol (Clifford et al., 2005; Fornos et al., 2006; Krass et al., 2007; Al Mazroui et al., 2009; Ali et al., 2012; Chan et al., 2012; Jarab et al., 2012; Mourão et al., 2013; Castejón et al., 2014; Wishah et al., 2015; Butt et al., 2016; Lim et al., 2016; Tourkmani et al., 2016; Korcegez et al., 2017; Shao et al., 2017) were finally included. Whereas, *n* = 1 study was not included in the final NWA, because it was the only study reporting for HDL, triglycerides and total cholesterol for intervention which involved diabetes education by health care professionals involving pharmacist as member (Kang et al., 2010).

When reference arm was set as usual care in the analysis, significant reduction in FBS [-31.89; 95% CI −42.31, −21.48; *p* < 0.001; *Z* = −6.00], SBP [−8.11; 95%CI −10.54, −5.69; *p* < 0.001; *Z* = −6.55], and, DBP [−3.19; 95% CI −4.79, −1.58; *p* < 0.001; *Z* = −3.89], BMI [−0.56; −1.01, −0.11; *p* = 0.014; −2.46], triglycerides [−0.41; 95%CI −0.60, −0.22; *p* < 0.001; *Z* = −4.23], and, low density lipoprotein [−0.36; 95%CI −0.50, −0.23; *p* < 0.001; *Z* = −5.14], was observed in the studies which involved pharmacist based diabetes education plus pharmaceutical care.

No additional benefit was observed for adding pharmaceutical care component of the intervention to pharmacist based educational intervention for BMI [0.05; 95%CI −0.48, 0.57], DBP [−1.56; 95%CI −3.81, 0.69], FBS [−6.01; 95%CI −20.59, 8.57], and LDL [−0.02; 95%CI −0.22, 0.19]. However, pharmacist based diabetes education plus pharmaceutical care was found to be statistically better than interventions involving pharmacist based diabetes education for lowering the levels of systolic blood pressure [−4.94; 95%CI −8.65, −1.23] and triglycerides [−0.26; 95%CI −0.51, −0.01].

Pharmacist based educational intervention without involving pharmaceutical care component were found to be effective for all secondary clinical outcomes [BMI, SBP, DBP, FBS, LDL], except for triglycerides [−0.15; 95%CI −0.32, 0.01; *p* = 0.073; *Z* = −1.8], high density lipoproteins [0.01; 95%CI −0.07, 0.10; *p* = 0.755; *Z* = 0.043], and, total cholesterol [−0.27; 95%CI −0.54, *p* = 0.054; *Z* = 0.139], in comparison to usual care. Similarly, such interventions were not found better than interventions which involved diabetes education by health care team involving pharmacist as member of team for SBP [1.16; 95%CI −4.54, 6.86], but were effective for BMI [−0.61; 95%CI −1.21, −0.02].

Studies were available to do NMA for only three secondary clinical outcomes (BMI, SBP and LDL) for the intervention which involved pharmacist as member of healthcare team. Statistically insignificant differences were observed for this intervention on systolic blood pressure [–4.94; 95%CI −9.30, 0.619; *p* = 0.086; *Z* = −1.72], BMI [0.00 95% CI −0.52, 0.53] and LDL [-0.06; 95%CI −0.38, 0.27] in comparison to usual care, details are presented in Table 2.

## Sensitivity analysis

Sensitivity analysis were performed to evaluate the efficacy on primary clinical outcome (HbA1c). In first sensitivity analysis four studies *n* = 4 studies (Odegard et al., 2005; Al Mazroui et al., 2009; Farsaei et al., 2011; Mehuys et al., 2011) which were showing selection bias, detection bias and other sources of bias, and were significantly contributing toward heterogeneity were excluded from the analysis. After excluding these studies the overall mean difference for HbA1c for *n* = 39 studies [−0.83; 95%CI −0.92, −0.75] remained same as including these studies. Similarly after removing these *n* = 4 studies, the mean difference for sub group analysis also remained the same (details are presented in Supplementary Table 3). Secondly, no significant reduction in the heterogeneity was observed while repeating all the analysis using fixed effect model (Supplementary Table 3).

## Discussion

This study is the first to present NMA on the comparative effect of various pharmacist based educational interventions in the management of people with type 2 diabetes, conducted globally, in different health care settings and using different experimental methodologies.

The studies included in this review involved educational and pharmaceutical care related interventions focusing on areas such as; diabetes and its complications, self-management and pharmaceutical care, directed at patients with type 2 diabetes. Evidence from the included studies suggest that pharmacist based interventions can have a clinically significant impact on glycemic control (HbA1c and FBS) and other clinical parameters, such as BMI, blood pressure and lipid profile of type 2 diabetes patients.

From the NMA it was revealed that there is no additional benefit of including pharmaceutical care component of intervention to the pharmacist based diabetes education on lowering the levels of HbA1c [−0.02; 95%CI −0.22, 0.17] and FBS [−6.01; 95%CI −20.59, 8.57] in type 2 diabetes patients. Similarly the interventions which involved pharmacist based diabetes education with pharmaceutical care [−0.13; 95%CI −0.47, 0.21] and without pharmaceutical care [−0.10; 95%CI −0.45, 0.24], were not statistically better in lowering HbA1c levels in comparison to the interventions which involved diabetes education provided by health care team involving pharmacist as team member. Interventions which involved pharmacist based diabetes education plus pharmaceutical care showed maximum beneficial effect on HbA1c levels, followed by pharmacist based diabetes education and diabetes education by HCT, as evident by SUCRA plot (Supplementary Figure 24).

These findings are similar to what were reported by systematic reviews conducted by Pousinho et al. (2016) and Van Eikenhorst et al. (2017), who reported clinically significant reductions in HbA1c levels, [−0.18% to −2.1%] and [−0.71%; 95% CI −0.91, −0.51] in type 2 diabetes patients, respectively. Systematic reviews done by Machado et al. (2007) and Yaghoubi et al. (2017) also reported comparable effects of pharmacist led interventions [−1.00 ± 0.28%; *p* < 0.001] and [0.96%; 95%CI 0.71, 1.22] respectively, when compared with usual care in diabetes patients.

In addition to HbA1c levels, the results of this NMA showed a statistically significant effect of pharmacist based diabetes education plus pharmaceutical care on most of the studied secondary clinical outcomes (FBS, BMI, SBP, DBP, LDL, HDL, and triglycerides) when compared to usual care. For secondary clinical outcomes, just like for glycemic control (HbA1c), there was no added value of pharmaceutical care component to the pharmacist based diabetes education intervention on FBS [−6.01; 95%CI −20.59, 8.57], BMI [0.05; 95%CI −0.48, 0.57], diastolic blood pressure [−1.56; 95%CI −3.81, 0.69], HDL [0.08; 95%CI −0.05, 0.20], total cholesterol [0.10; 95%CI −0.29, 0.49], and, LDL [−0.02; 95%CI −0.22, 0.19] of type 2 diabetes patients. However, pharmacist based diabetes education plus pharmaceutical care was significantly better than pharmacist based diabetes education for SBP [−4.94; 95%CI −8.65, −1.23] and triglycerides [−0.26; 95%CI −0.51, −0·01]. Likewise, such interventions were significantly better than diabetes education by health care team for BMI [−0.57; 95%CI −1.25, −0.12].

It is evident from the NMA of this review that pharmacist based diabetes education plus pharmaceutical care interventions were associated with additional clinical benefits beyond glycemic control and these included improvement in systolic blood pressure and triglycerides levels. Tight glycaemic control along with blood pressure control could be of clinical significance in reducing the incidence of complications associated with type 2 diabetes. In United Kingdom prospective diabetes study (UKPDS), there was reductions in diabetes related complications (12%), diabetes associated mortality (15%), myocardial infarctions (11%) and microvascular complications (13%), with each 10 mmHg decrease in systolic blood pressure (Adler et al., 2000).

Interventions involving pharmacist based diabetes education were comparable in effect to pharmacist based education plus pharmaceutical care for glycemic control (HbA1c and FBS) and many of the secondary clinical outcomes (BMI, DBP, HDL, TC, and LDL). In comparison to diabetes education by health care team, pharmacist based diabetes education was not significantly batter for SBP [1.16; 95%CI −4.54, 6.86] and LDL [−0.31; 95%CI −0.66, 0.05], except for BMI [−0.57; 95%CI −1.25, −0.12].

For diabetes education by health care team including pharmacist, studies were available to do NMA for HbA1c and few of the secondary clinical outcomes (BMI, SBP, and LDL). It was observed from the NMA that efficacy of diabetes education by health care team was comparable to other studied interventions for HbA1c and systolic blood pressure, but was not significantly better than usual care for BMI [0.00; 95%CI −0.52, 0.53] and LDL [−0.06; 95%CI −0.38, 0.27].

According to the literature, other interventions, such as involving self-management and behavioral education interventions delivered by a diverse group of healthcare providers also demonstrated clinically significant reductions in HbA1c levels. Chrvala et al. (2016) conducted a systematic review to estimate the impact of diabetes self-management education in type 2 diabetes patients by a diversified group of healthcare professionals. Among the 118 included studies, Chrvala et al. (2016) found that HbA1c levels were reduced with an average of −0.74%, which is comparable to our NMA findings of A1c levels [−0.73%; 95%CI −1.036, −0.414], when diabetes education was provided by health care team, which involved pharmacist as a team member. This was similarly noted in another study in which a healthcare team approach, which was delivered remotely could reduce HbA1c levels by 0.58% (Lee et al., 2017).

Findings of our NMA show that pharmacist based interventions, irrespective of the nature of the intervention, have shown clinically significant reductions in HbA1c levels ranging from 0.72 to 0.86% when compared to usual care. These reductions are of clinical importance, as according to literature there is 25% reduction in microvascular complications and 10% reduction in diabetes related mortality, with every 1% decrease in HbA1c level, which eventually result in healthcare cost reductions and improved patients quality of life (Palmer et al., 2004). However, our NMA showed that the pharmacist based interventions which imparted education along with pharmaceutical care, had an additional significantly better effect on SBP, triglycerides levels (in comparison to pharmacist based diabetes education), and BMI (diabetes education by HCT). Although tight glycemic control is critical for diabetes patients, but blood pressure control is also important for reducing the risk of cardiovascular disease (Adler et al., 2000; El-Shafie and Rizvi, 2010). The finding of this NMA will facilitate policy makers in formulating or selecting among the different available interventions, keeping in view the desired beneficial outcomes and available healthcare resources.

As pharmacists are easily accessible among rest of the health care professionals, and in addition, their knowledge about diabetes pharmacotherapy and self-management skills make them unique for helping the type 2 diabetes patients who cannot be managed with existing healthcare system, either due to unaffordability and/or inaccessibility to healthcare facilities (Lee and Mak, 2017). This fact of larger impact of pharmacist based self-management education is supported by Sherifali et al. (2015). The results of our NMA demonstrates the clinical impact of diabetes self-management education in adult type 2 diabetes patients. The interventions which involved diabetes education in combination with pharmaceutical care was the best intervention as compared to the other interventions included in our NMA. Overall among the *n* = 39 RCTs which were included in this NMA, selection, detection and performance bias were < 25%, which could possibly influence the NMA results, therefore, it's recommended to interpret the results of our NMA with caution. Due to poor content elaboration and varied nature of pharmaceutical care interventions, it was extremely difficult to comment which type of intervention will be the most effective in combination with diabetes self-management education. This is a common issue especially in complex interventions, where the description of methods are usually insufficient to extract and tease out the important elements which may contribute to the success of the program. As such, it is recommended that a separate protocol be published to enable readers and other researchers to better identify and understand the study elements so that this can be replicated in the future.

Further research will be needed to evaluate the interventions with respect to time, frequency and contents of the pharmaceutical care intervention, so that we can get defined and better outcomes. In addition, this study furnishes important insights for future research focusing a tailored intervention and investigation of cost involved in delivering such interventions, to design cost effective interventions. The outcomes of which will help policy makers in selection of suitable interventions keeping in view the best utility of the available resources.

## Strengths and limitations

There are several limitations to this study. There were fewer studies in one subgroup “diabetes education by health care team, involving pharmacist as a member,” made it difficult to get a clearer picture of the comparison. Secondly, there was high heterogeneity in the pooled analysis, which warrant interpretation with care, this variation could be due varied sample size of the trials, population characteristics, difference in study designs, and nature of intervention applied. This issue was resolved to some extents after performing sub-group analysis and removing the poor quality studies from the NMA, which were significantly contributing to heterogeneity. Lastly, due to diversified nature of the pharmaceutical care contents along with diabetes education, all such pharmaceutical care based interventions were classified into one category.

## Conclusion

This review demonstrates a comprehensive evidence for clinically beneficial impact of pharmacist based interventions on glycaemic control and other clinical outcomes of type 2 diabetes patients.

No intervention appeared to be better than the other for the primary outcome (HbA1c). Although tight glycaemic control is of immense importance for diabetes patients, but the reduction in blood pressure and lipid control is equally important in diabetic patients and is associated with reduction in the risk of cardiovascular disease in diabetic patients. Diabetes education along with pharmaceutical care was statistically significant in lowering systolic blood pressure and triglycerides levels, as compared to rest of the interventions. Although, pharmacists are involved in a variety of interventions, varying from diabetes education, self-management, alone, or in combination with pharmaceutical care planning, yet, an overall positive effect on metabolic control has been observed. The evidence synthesized from this this study could be of significant value for health policy makers in selecting ideal intervention for diabetes self-management education for type 2 diabetes patients keeping in view the available health resources.

## Author contributions

AB and TK: Conceived the idea and perform the initial search that was validated by L-HL and K-GC; AB: Wrote the initial draft, which was finalized by TK, SL, K-GC, and L-HL. TK, SL, and AB: Did the statistical analysis; SL and TK: Proof read final manuscript draft for correction. All authors equally contributed to this project.

### Conflict of interest statement

The authors declare that the research was conducted in the absence of any commercial or financial relationships that could be construed as a potential conflict of interest.
