# Supplementary material for: Efficacy of Pharmacist Based Diabetes Educational Interventions on Clinical Outcomes of Adults With Type 2 Diabetes Mellitus: A Network Meta-Analysis
Source: Front Pharmacol. 2018 Apr 10;9:339. doi: 10.3389/fphar.2018.00339 (PMC5902757; doi:10.3389/fphar.2018.00339)
Supplement: Supplementary file 1 [file DataSheet1.DOCX]

Effectiveness of pharmacist based diabetes educational interventions on glycaemic control of adults with type 2 diabetes mellitus: Systematic review and network meta-analysis

Allah Bukhsh, Tahir Mehmood Khan, Shaun Wen Huey Lee, Learn-Han Lee, Kok-Gan Chan, Bey-Hing Goh

Supplementary material: Contents

Appendix 1 Search strategy

Supplementary Table 1: Reasons for study exclusion after full-text assessment (n=426)

Supplementary Table 2: Details of pharmaceutical care intervention contents delivered to the participants in provided in combination with diabetes education

Supplementary Table 3: Results of subgroup and sensitivity analysis conducted for primary clinical outcome (HbA1c)

Supplementary Figure 1: Overall meta-analysis for HbA1c of included RCTs (n=43)

Supplementary Figure 2: Sub-group meta-analysis for HbA1c of included RCTs (n=43)

Supplementary Figure 3: Sub-group meta-analysis for HbA1c of studies after removing studies which were contributing significant heterogeneity (n=39)

Supplementary Figure 4: Overall meta-analysis for BMI of included RCTs (n=20)

Supplementary Figure 5: Sub-group meta-analysis for BMI of included RCTs (n=20)

Supplementary Figure 6: Sub-group meta-analysis for BMI of included RCTs (n=15) after removing 5 studies which were significantly contributing towards heterogeneity

Supplementary Figure 7: Overall meta-analysis for FBS of included RCTs (n=16)

Supplementary Figure 8: Sub-group meta-analysis for FBS of included RCTs (n=16)

Supplementary Figure 9: Sub-group meta-analysis for FBS of included RCTs (n=14) after removing 2 studies which were significantly contributing towards heterogeneity

Supplementary Figure 10: Overall meta-analysis for SBP of included RCTs (n=19)

Supplementary Figure 11: Sub-group meta-analysis for SBP of included RCTs (n=19)

Supplementary Figure 12: Sub-group meta-analysis for SBP of included RCTs (n=18) after removing 1 study which was significantly contributing towards heterogeneity

Supplementary Figure 13: Overall meta-analysis for DBP of included RCTs (n=17)

Supplementary Figure 14: Sub-group meta-analysis for SBP of included RCTs (n=17)

Supplementary Figure 15: Sub-group meta-analysis for DBP of included RCTs (n=15) after removing 2 studies which were significantly contributing towards heterogeneity

Supplementary Figure 16: Overall meta-analysis for LDL of included RCTs (n=18)

Supplementary Figure 17: Sub-group meta-analysis for LDL of included RCTs (n=18)

Supplementary Figure 18: Overall meta-analysis for triglycerides of included RCTs (n=16)

Supplementary Figure 19: Sub-group meta-analysis for triglycerides of included RCTs (n=16)

Supplementary Figure 20: Overall meta-analysis for total cholesterol of included RCTs (n=16)

Supplementary Figure 21: Sub-group meta-analysis for total cholesterol of included RCTs (n=16)

Supplementary Figure 22: Overall meta-analysis for high density lipoprotein of included RCTs (n=15)

Supplementary Figure 23: Sub-group meta-analysis for high density lipoprotein of included RCTs (n=15)

Supplementary Figure 24: Surface under cumulative ranking curve of the various interventions for glycosylated hemoglobin.

**APPENDIX I: SEARCH STRATEGY**

**Search Term**

("Diabetes Mellitus, Type 2" OR T2DM OR "Non insulin dependent diabetes mellitus “OR "NIDDM" OR "Type 2 diabetes")

**AND**

("Pharmaceutical care" OR "Clinical pharmacy" OR "Community pharmacy" OR Pharmacist* OR “Pharmaceutical services" OR Education OR Intervention* OR "Self care" OR "Self-management" OR "Medication Management")

**AND**

(Knowledge OR "Hemoglobin A, Glycosylated" OR "HbA1c" OR "glyc?emic control" OR "Behavio?r change")

**PubMed**

Diabetes Mellitus, Type 2 [MeSH) 118965

T2DM 12990

Non insulin dependent diabetes mellitus 129901

NIDDM 119945

Type 2 diabetes 143955

("Diabetes Mellitus, Type 2" OR T2DM OR "Non insulin dependent diabetes mellitus " OR "NIDDM" OR "Type 2 diabetes") 146323

Pharmaceutical care 86464

Clinical pharmacy 71440

Community pharmacy 22905

Pharmacist* [MeSH] 30280

Pharmaceutical services [MeSH] 70152

Education 1169774

Intervention* 866222

Self care [MeSH] 155618

Self-management 180204

Medication management 86929

("Pharmaceutical care" OR "Clinical pharmacy" OR "Community pharmacy" OR Pharmacist* OR "Pharmaceutical services" OR Education OR Intervention* OR "Self care" OR "Self-management" OR "Medication Management") 1946331

Hemoglobin A, Glycosylated [MeSH] 28468

Behavior change 132814

Behaviour change 17292

HbA1c 41978

glycemic control 22282

glycaemic control 8019

Knowledge [MeSH] 607197

(Knowledge OR "Hemoglobin A, Glycosylated" OR "HbA1c" OR "glycemic control" OR "Behavior change" OR "glycaemic control" OR "Behaviour change") 670612

("Diabetes Mellitus, Type 2" OR T2DM OR "Non insulin dependent diabetes mellitus " OR "NIDDM" OR "Type 2 diabetes")

AND ("Pharmaceutical care" OR "Clinical pharmacy" OR "Community pharmacy" OR Pharmacist* OR "Pharmaceutical services" OR Education OR Intervention* OR "Self care" OR "Self-management" OR "Medication Management") AND (Knowledge OR "Hemoglobin A, Glycosylated" OR "HbA1c" OR "glycemic control" OR "Behavior change" OR "glycaemic control" OR "Behaviour change") **7902**

**Ovid Medline**

Diabetes Mellitus, Type 2 114017

T2DM 10427

Non insulin dependent diabetes mellitus 7026

NIDDM 6969

Type 2 diabetes 84298

("Diabetes Mellitus, Type 2" OR T2DM OR "Non insulin dependent diabetes mellitus " OR "NIDDM" OR "Type 2 diabetes") 137179

Pharmaceutical care 1626

Clinical pharmacy 2763

Community pharmacy 4435

Pharmacist* [MeSH] 26247

Pharmaceutical services [MeSH] 11920

Education 645806

Intervention* 720674

Self care [MeSH] 37016

Self-management 11485

Medication management 1978

("Pharmaceutical care" OR "Clinical pharmacy" OR "Community pharmacy" OR Pharmacist* OR "Pharmaceutical services" OR Education OR Intervention* OR "Self care" OR "Self-management" OR "Medication Management") 1346219

Hemoglobin A, Glycosylated [MeSH] 30476

Behavior change 6225

Behaviour change 2821

HbA1c 20766

glycemic 16749

glycaemic control 6551

Knowledge [MeSH] 535740

(Knowledge OR "Hemoglobin A, Glycosylated" OR "HbA1c" OR "glycemic control" OR "Behavior change" OR "glycaemic control" OR "Behaviour change") 533679

("Diabetes Mellitus, Type 2" OR T2DM OR "Non insulin dependent diabetes mellitus " OR "NIDDM" OR "Type 2 diabetes")

AND ("Pharmaceutical care" OR "Clinical pharmacy" OR "Community pharmacy" OR Pharmacist* OR "Pharmaceutical services" OR Education OR Intervention* OR "Self care" OR "Self-management" OR "Medication Management") AND (Knowledge OR "Hemoglobin A, Glycosylated" OR "HbA1c" OR "glycemic control" OR "Behavior change" OR "glycaemic control" OR "Behaviour change") **6786**

**ProQuest**

Diabetes Mellitus, Type 2 50543

T2DM 6034

Non insulin dependent diabetes mellitus 3355

NIDDM 1463

Type 2 diabetes 76212

("Diabetes Mellitus, Type 2" OR T2DM OR "Non insulin dependent diabetes mellitus " OR "NIDDM" OR "Type 2 diabetes") 59204

Pharmaceutical care 20678

Clinical pharmacy 12692

Community pharmacy 5019

Pharmacist* 17312

Pharmaceutical services 9163

Education 1563757

Intervention* 673316

Self care 94173

Self-management 13366

Medication management 22408

("Pharmaceutical care" OR "Clinical pharmacy" OR "Community pharmacy" OR Pharmacist* OR "Pharmaceutical services" OR Education OR Intervention* OR "Self care" OR "Self-management" OR "Medication Management") 2151245

Hemoglobin A, Glycosylated 7466

Behavior change 305186

Behaviour change 305186

HbA1c 10975

glycemic control 14281

glycaemic control 14281

Knowledge 821893

(Knowledge OR "Hemoglobin A, Glycosylated" OR "HbA1c" OR "glycemic control" OR "Behavior change" OR "glycaemic control" OR "Behaviour change") 858837

("Diabetes Mellitus, Type 2" OR T2DM OR "Non insulin dependent diabetes mellitus “OR "NIDDM" OR "Type 2 diabetes")

AND ("Pharmaceutical care" OR "Clinical pharmacy" OR "Community pharmacy" OR Pharmacist* OR “Pharmaceutical services" OR Education OR Intervention* OR "Self care" OR "Self-management" OR "Medication Management") AND (Knowledge OR "Hemoglobin A, Glycosylated" OR "HbA1c" OR "glycemic control" OR "Behavior change" OR "glycaemic control" OR "Behaviour change") **5767**

**EBSCOhost (CINAHL Plus & Medline)**

Diabetes Mellitus, Type 2

T2DM

Non insulin dependent diabetes mellitus

NIDDM

Type 2 diabetes

("Diabetes Mellitus, Type 2" OR T2DM OR "Non insulin dependent diabetes mellitus “OR "NIDDM" OR "Type 2 diabetes")

Pharmaceutical care

Clinical pharmacy

Community pharmacy

Pharmacist*

Pharmaceutical services

Education

Intervention*

Self care

Self-management

Medication management

("Pharmaceutical care" OR "Clinical pharmacy" OR "Community pharmacy" OR Pharmacist* OR “Pharmaceutical services" OR Education OR Intervention* OR "Self care" OR "Self-management" OR "Medication Management")

Hemoglobin A, Glycosylated

Behavior change

Behaviour change

HbA1c

glycemic control

glycaemic control

Knowledge

(Knowledge OR "Hemoglobin A, Glycosylated" OR "HbA1c" OR "glycemic control" OR "Behavior change" OR "glycaemic control" OR "Behaviour change")

("Diabetes Mellitus, Type 2" OR T2DM OR "Non insulin dependent diabetes mellitus “OR "NIDDM" OR "Type 2 diabetes")

**AND** ("Pharmaceutical care" OR "Clinical pharmacy" OR "Community pharmacy" OR Pharmacist* OR "Pharmaceutical services" OR Education OR Intervention* OR "Self care" OR "Self-management" OR "Medication Management") **AND** (Knowledge OR "Hemoglobin A, Glycosylated" OR "HbA1c" OR "glycemic control" OR "Behavior change" OR "glycaemic control" OR "Behaviour change") **2149**

**SCOPUS**

Diabetes Mellitus, Type 2

T2DM

Non insulin dependent diabetes mellitus

NIDDM

Type 2 diabetes

("Diabetes Mellitus, Type 2" OR T2DM OR "Non insulin dependent diabetes mellitus " OR "NIDDM" OR "Type 2 diabetes")

Pharmaceutical care

Clinical pharmacy

Community pharmacy

Pharmacist*

Pharmaceutical services

Education

Intervention*

Self care

Self-management

Medication management

("Pharmaceutical care" OR "Clinical pharmacy" OR "Community pharmacy" OR Pharmacist* OR "Pharmaceutical services" OR Education OR Intervention* OR "Self care" OR "Self-management" OR "Medication Management")

Hemoglobin A, Glycosylated

Behavior change

Behaviour change

HbA1c

glycemic control

glycaemic control

Knowledge

(Knowledge OR "Hemoglobin A, Glycosylated" OR "HbA1c" OR "glycemic control" OR "Behavior change" OR "glycaemic control" OR "Behaviour change")

("Diabetes Mellitus, Type 2" OR T2DM OR "Non insulin dependent diabetes mellitus " OR "NIDDM" OR "Type 2 diabetes")

**AND** ("Pharmaceutical care" OR "Clinical pharmacy" OR "Community pharmacy" OR Pharmacist* OR "Pharmaceutical services" OR Education OR Intervention* OR "Self care" OR "Self-management" OR "Medication Management") **AND** (Knowledge OR "Hemoglobin A, Glycosylated" OR "HbA1c" OR "glycemic control" OR "Behavior change" OR "glycaemic control" OR "Behaviour change") **7286**

**Supplementary Table 1: Reasons for study exclusion after full-text assessment (n=426)**

| **Database** | **Number of records** |
| --- | --- |
| Editorial / letter to editor | **7** |
| Study protocol | **2** |
| Review articles | **18** |
| Conference paper / proceedings  Non RCT studies | **22**  **25** |
| Does not meet selection criteria (details are as under)- | **352** |
| *Did not measure HbA1c* | *57* |
| *Observed impact of diabetes education provided by Physicians,*  *HCW, Dietitian, nutritionist, diabetes educator (no pharmacist)* | *122* |
| *Diabetes education mainly by nurses (no pharmacist)* | *71* |
| *Diabetes education by internet technology, mobile text*  *messaging, internet (no pharmacist involved)* | *22* |
| *Telemedicine (no pharmacist)* | *4* |
| *Qualitative studies* | *13* |
| *No diabetes education / pharmaceutical intervention without education / Measured effect of different hypoglycemic agents on HbA1c* | *42* |
| *Measured impact of education on cost, QoL, curriculum or policy design* | *21* |

Hba1c = Glycosylated haemoglobin; QoL = Quality of life; HCW = Health care worker; RCT = Randomized control trial.

**Supplementary Table 2: Details of pharmaceutical care intervention contents delivered to the participants in combination with diabetes education**

| **Authors, year, country** | **Pharmaceutical care intervention** | **Details** |
| --- | --- | --- |
| Ali et al. 2012, United Kingdom | Pharmaceutical care package | Patient tailored medication review. |
| Nascimentoa, 2016, Spain | Individualized pharmacotherapy management service | Analysis of necessity, safety and effectiveness of medications. |
| Wishah et al. 2015, Jordan | Focused care plans | Therapeutic efficacy monitoring by laboratory results.  Recommendations for initiation of oral hypoglycemic agents, titration of drug therapeutic dosage, and current therapy modifications due to ineffectiveness. |
| Taylor et al. 2005, | Medication review | Complete medication regimen review.  Documentation of all the findings and recommendations for each patient. |
| Clifford et al. 2005, Australia | Pharmaceutical care program. | Patient tailored pharmaceutical care plan designing and implementation.  Listing and ranking drug-related problems;  Establishing pharmacotherapeutic outcomes with the patient.  Developing feasible pharmacotherapeutic alternatives.  Selection of the best pharmacotherapeutic agent(s) |
| Jacobs et al. 2012, USA | Comprehensive medication review | Medications review of prescription, over-the-counter products, herbal  remedies, and dietary supplements  Targeted physical assessment including weight, height, blood pressure, pulse, and foot exam. |
| Mahwi and Obied 2013, Iraq | Pharmaceutical care program | Evaluation of drug therapy problems and compliance by pill count and medication adherence tool. |
| Moura˜o et al. 2013, Brazil | Pharmaceutical care program | Identification of drug therapy problems.  Individualized Pharmacotherapy care plan, including patient education and / or pharmacotherapy changes. |
| Rothman et al.2005, USA | Disease management program | Recommendation for therapy initiation.  Devising algorithms for insulin titration and metformin. |
| Cohen et al. 2011, USA | Diabetes care plan (DCP) | Identification and resolution of medication-related problems, requiring intervention. |
| Ahmad et al. 2015, Sudan | Pharmacist-led pharmaceutical care | Drug therapy Optimization. |
| Doucette et al. 2009, USA | Extended Diabetes Care | Medication review.  Identification of therapy related issues.  Recommendations for therapy modification. |
| Chung et al. 2014, Malaysia | Pharmaceutical care model | Medications review.  Resolution of medication related problems. |
| Scott et al. 2006, USA | Pharmacist-managed diabetes care | Pharmacotherapeutic consultations. |
| Fornos et al. 2006, Spain | Pharmacotherapy follow-up program | Identification, prevention and resolution of drug therapy related problems. |
| Jameson, et al.2010, USA | Pharmacist management of diabetes | Medication management. |
| Armour et al. 2004, Australia | Community Pharmacist delivered interventions | Conducting medication checks and evaluation. |
| Ko et al. 2017, USA | Medication Management Program | Evaluation of patient’s therapy needs.  Patient tailored care planning. |
| Korcegez et al. 2017, USA | Medication and treatment review. | Discussion sessions on revision of medications. |
| Chen et al. 2016, Taiwan | Pharmaceutical care of elderly patients | Assessment of adherence to pillbox use and insulin injection technique.  Evaluation for appropriateness of current medication regimens.  Suggesting modifications to medication regimens to patients’ physicians. |
| Krass et al. 2007, Sydney | Continuity-of-care model | Detection of drug-related problems and appropriate physician referrals. |

Supplementary Table 3: Results of subgroup and sensitivity analysis conducted for primary clinical outcome (HbA1c)

| **_Study characteristics_** | | | | **_Without removing studies which were contributing to significant heterogeneity_** | | | | **_After removing studies (n=4) which were contributing to significant heterogeneity_** | | | | | |
| --- | --- | --- | --- | --- | --- | --- | --- | --- | --- | --- | --- | --- | --- |
| **_Intervention_** | **_Subgroup_** | **_No of studies_** | **_Sample size_** | **_Fixed effects (95% CI)_** | **_I_^2^** | **_Random effects (95% CI)_** | **_I_^2^ _; Tau_^2^ _; p value_** | **_No of studies_** | **_Sample size_** | **_Fixed effects (95% CI)_** | **_I_^2^** | **_Random effects (95% CI)_** | **_I_^2^ _; Tau_^2^ _; p value_** |
| _Education_ |  | _43_ | _6259_ | _-0.85 [-0.89, -0.81]_ | _79%_ | _-0.85 [-0.96, -0.75]_ | _79%; 0.08; <0.0001_ | _39_ | _5534_ | _-0.88 [-0.93, -0.84]_ | _57%_ | _-0.83 [-0.92,-0.75]_ | _57%; 0.03; <0.00001_ |
|  | _Pharm-led edu_ | _17_ | _2679_ | _-0.87 [-0.92, -0.82]_ | _87%_ | _-0.90 [-1.07, -0.74]_ | _87%; 0.08; <0.00001_ | _14_ | _2020_ | _-0.92 [-0.98, -0.86]_ | _43%_ | _-0.85 [-0.95, -0.75]_ | _43%; 0.01; <0.00001_ |
|  | _Pharm-led edu + PC_ | _22_ | _2794_ | _-0.81[ -0.89, -0.72]_ | _68%_ | _-0.83 [-0.98, -0.67]_ | _68%; 0.09; <0.00001_ | _21_ | _2828_ | _-0.83 [-0.92, -0.74]_ | _63%_ | _-0.86 [-1.01, -0.71]_ | _63%; 0.07; <0.00001_ |
|  | _Pharm +HCT_ | _4_ | _786_ | _-0.70 [-0.91, -0.48]_ | _37%_ | _-0.72 [-1.02, -0.43]_ | _37%; 0.03; 0.19_ | _4_ | _786_ | _-0.70 [-0.91, -0.48]_ | _37%_ | _-0.72 [-1.02, -0.43]_ | _37%; 0.03; <0.00001_ |
| _Study Location_ |  | _43_ | _6259_ | _-0.85 [-0.89, -0.81]_ | _79%_ | _-0.85 [-0.96, -0.75]_ | _79%; 0.08; <0.0001_ | _39_ | _5534_ | _-0.88 [-0.93, -0.84]_ | _57%_ | _-0.83 [-0.92,-0.75]_ | _57%; 0.03; 0.0001_ |
|  | _Asia_ | _20_ | _3398_ | _-1.0 [-1.05, -0.95]_ | _76%_ | _-1.02 [-1.16, -0.88]_ | _76%; 0.06; <0.00001_ | _18_ | _3019_ | _-0.95 [-1.01, -0.90]_ | _58%_ | _-0.93 [-1.05, -0.82]_ | _58%; 0.03; <0.00001_ |
|  | _North America_ | _11_ | _1192_ | _-0.75 [-0.86 ,-0.63]_ | _57%_ | _-0.73 [-0.92, -0.54]_ | _57%; 0.05; <0.00001_ | _10_ | _1126_ | _-0.78 [-0.90, -0.67]_ | _30%_ | _-0.79 [-0.94, -0.65]_ | _30%; 0.02; <0.00001_ |
|  | _Europe_ | _5_ | _589_ | _-0.55 [-0.65, -0.45]_ | _52%_ | _-0.70 [-0.94, -0.46]_ | _52%; 0.03; 0.00001_ | _4_ | _309_ | _-0.80 [-1.03, -0.56]_ | _7%_ | _-0.80 [-1.05, -0.55]_ | _7%; 0.01; 0.36_ |
|  | _Australia_ | _4_ | _610_ | _-0.56 [-0.72, -0.39]_ | _0%_ | _-0.56 [-0.72, -0.39]_ | _0%; 0.00; 0.64_ | _4_ | _610_ | _-0.56 [-0.72, -0.39]_ | _0%_ | _-0.56 [-0.72, -0.39]_ | _0%; 0.00; <0.00001_ |
|  | _South America_ | _2_ | _170_ | _-0.86 [-1.32, -0.40]_ | _66%_ | _-0.88 [-1.68, -0.09]_ | _66%; 0.22; 0.09_ | _2_ | _170_ | _-0.86 [-1.32, -0.40]_ | _66%_ | _-0.88 [-1.68, -0.09]_ | _66%; 0.22; 0.09_ |
|  | _Africa_ | _1_ | _300_ | _-0.60 [-0.98, -0.22]_ | _ND_ | _-0.60 [-0.98, -0.22]_ | _ND_ | _1_ | _300_ | _-0.60 [-0.93, -0.84]_ | _ND_ | _-0.60 [-0.98, -0.22]_ | _ND_ |
| _Baseline HbA1c of the_  _study participants_ |  |  |  |  |  |  |  |  |  |  |  |  |  |
|  | _<8.0%_ | _17_ | _2409_ | _-0.63 [-0.70, -0.55]_ | _61%_ | _-0.72 [-0.87, -0.58]_ | _61%; 0.05; <0.00001_ | _1_ | _2139_ | _-0.75 [-0.85, -0.64]_ | _51%_ | _-0.75 [-0.91, -0.60]_ | _51%; 0.05; 0.010_ |
|  | _>8.0%_ | _26_ | _2850_ | _-0.95 [-1.00, -0.90]_ | _76%_ | _-0.93 [-1.06, -0.80]_ | _76%; 0.06; <0.00001_ | _3_ | _2395_ | _-0.92 [-0.97, -0.86]_ | _55%_ | _-0.88 [-0.92, -0.78]_ | _55%; 0.02; 0.0009_ |
| _Duration of intervention_ |  |  |  |  |  |  |  |  |  |  |  |  |  |
|  | _< 6 months_ | _7_ | _929_ | _-0.94 [-1.02, -0.85]_ | _85%_ | _1.02 [-1.32, -0.71]_ | _85%; 0.12; <0.00001_ | _1_ | _784_ | _-0.90 [-0.99, -0.81]_ | _74%;_ | _-0.85 [-1.11, -0.60]_ | _74%; 0.06; <0.00001_ |
|  | _≥ 6 months_ | _36_ | _5330_ | _-0.82 [-0.87, -0.78]_ | _77%_ | _-0.82 [-0.94, -0.70]_ | _77%: 0.08: <0.00001_ | _3_ | _4750_ | _-0.88 [-0.93, -0.82]_ | _53%_ | _-0.83 [-0.93, -0.74]_ | _53%; 0.03; <0.00001_ |

Pharm Led DMEDU +PC = Pharmacist based diabetes education plus pharmaceutical care; Pharm Led DMEDU= Pharmacist based diabetes education; DMEDU Pharm + HCT = Diabetes education by health care team involving pharmacist as member; ND = no data.

**Supplementary Figure 1: Overall meta-analysis for HbA1c of included RCTs (n=43)**

**Supplementary Figure 2: Sub-group meta-analysis for HbA1c of included RCTs (n=43)**

**Supplementary Figure 3: Sub-group meta-analysis for HbA1c of studies after removing studies which were contributing significant heterogeneity (n=39)**

**Supplementary Figure 4: Overall meta-analysis for BMI of included RCTs (n=20)**

**Supplementary Figure 5: Sub-group meta-analysis for BMI of included RCTs (n=20)**

**Supplementary Figure 6: Sub-group meta-analysis for BMI of included RCTs (n=15) after removing 5 studies which were significantly contributing towards heterogeneity**

**Supplementary Figure 7: Overall meta-analysis for FBS of included RCTs (n=16)**

**Supplementary Figure 8: Sub-group meta-analysis for FBS of included RCTs (n=16)**

**Supplementary Figure 9: Sub-group meta-analysis for FBS of included RCTs (n=14) after removing 2 studies which were significantly contributing towards heterogeneity**

**Supplementary Figure 10: Overall meta-analysis for SBP of included RCTs (n=19)**

**Supplementary Figure 11: Sub-group meta-analysis for SBP of included RCTs (n=19)**

**Supplementary Figure 12: Sub-group meta-analysis for SBP of included RCTs (n=18) after removing 1 study which was significantly contributing towards heterogeneity**

**Supplementary Figure 13: Overall meta-analysis for DBP of included RCTs (n=17)**

**Supplementary Figure 14: Sub-group meta-analysis for SBP of included RCTs (n=17)**

**Supplementary Figure 15: Sub-group meta-analysis for DBP of included RCTs (n=15) after removing 2 studies which were significantly contributing towards heterogeneity**

**Supplementary Figure 16: Overall meta-analysis for LDL of included RCTs (n=18)**

**Supplementary Figure 17: Sub-group meta-analysis for LDL of included RCTs (n=18)**

**Supplementary Figure 18: Overall meta-analysis for triglycerides of included RCTs (n=16)**

**Supplementary Figure 19: Sub-group meta-analysis for triglycerides of included RCTs (n=16)**

**Supplementary Figure 20: Overall meta-analysis for total cholesterol of included RCTs (n=16)**

**Supplementary Figure 21: Sub-group meta-analysis for total cholesterol of included RCTs (n=16)**

**Supplementary Figure 22: Overall meta-analysis for high density lipoprotein of included RCTs (n=15)**

**Supplementary Figure 23: Sub-group meta-analysis for high density lipoprotein of included RCTs (n=15)**

**Supplementary Figure 24: Surface under cumulative ranking curve of the various interventions for glycosylated hemoglobin.**

**Ranking based upon SUCRA plot**

1. Edu + PC: Pharmacist based diabetes education plus pharmaceutical care
2. Edu: Pharmacist based diabetes education
3. HC: Diabetes education by health care team involving pharmacist as member
4. UC: Usual care
